# Planning and implementing genetic rescue of an endangered freshwater fish population in a regulated river, where low flow reduces breeding opportunities and may trigger inbreeding depression

**DOI:** 10.1111/eva.13679

**Published:** 2024-04-11

**Authors:** Alexandra Pavlova, Nadja M. Schneller, Mark Lintermans, Matt Beitzel, Diana A. Robledo‐Ruiz, Paul Sunnucks

**Affiliations:** ^1^ School of Biological Sciences Monash University Melbourne Victoria Australia; ^2^ Centre for Applied Water Science Institute for Applied Ecology, University of Canberra Canberra Australian Capital Territory Australia; ^3^ Environment, Planning & Sustainable Development Directorate (ACT Government) Canberra Australian Capital Territory Australia

**Keywords:** genetic diversity, genetic management, genetic rescue, inbreeding depression, Macquarie perch *Macquaria australasica*, native Australian wildlife, population persistence, translocations

## Abstract

Augmenting depleted genetic diversity can improve the fitness and evolutionary potential of wildlife populations, but developing effective management approaches requires genetically monitored test cases. One such case is the small, isolated and inbred Cotter River population of an endangered Australian freshwater fish, the Macquarie perch *Macquaria australasica*, which over 3 years (2017–2019) received 71 translocated migrants from a closely related, genetically more diverse population. We used genetic monitoring to test whether immigrants bred, interbred with local fish and augmented population genetic diversity. We also investigated whether levels of river flow affected recruitment, inbreeding depression and juvenile dispersal. Fish length was used to estimate the age, birth year cohort and growth of 524 individuals born between 2016 and 2020 under variable flow conditions. DArT genome‐wide genotypes were used to assess individual ancestry, heterozygosity, short‐term effective population size and identify parent‐offspring and full‐sibling families. Of 442 individuals born after translocations commenced, only two (0.45%) were of mixed ancestry; these were half‐sibs with one translocated parent in common. Numbers of breeders and genetic diversity for five birth year cohorts of the Cotter River fish were low, especially in low‐flow years. Additionally, individuals born in the year of lowest flow evidently suffered from inbreeding depression for juvenile growth. The year of highest flow was associated with the largest number of breeders, lowest inbreeding in the offspring and greatest juvenile dispersal distances. Genetic diversity decreased in the upstream direction, flagging restricted access of breeders to the most upstream breeding sites, exacerbated by low river flow. Our results suggest that the effectiveness of translocations could be increased by focussing on upstream sites and moving more individuals per year; using riverine sources should be considered. Our results indicate that river flow sufficient to facilitate fish movement through the system would increase the number of breeders, promote individuals' growth, reduce inbreeding depression and promote genetic rescue.

## 1 INTRODUCTION

Genetic diversity underlies the fitness and evolutionary potential of wildlife populations and hence is increasingly considered in planning biodiversity conservation (Liddell et al., 2021). Genetic management sets out practical paths to detect and address serious genetic problems, but test cases with carefully monitored outcomes are required to optimize management recommendations across a broad range of specific cases (Frankham et al., 2019).

Healthy self‐sustaining populations have high genetic diversity, which provides material for evolving adaptation to changes in the environment (Hoffmann & Sgrò, 2011). Small populations isolated from larger gene pools through anthropogenic habitat modifications rapidly lose genetic diversity by genetic drift. This elevates their risk of extinction through lowered capacity to adapt to changing environments, inbreeding and resulting loss of fitness (inbreeding depression; Frankham et al., 2017). Inbreeding depression is typically expressed to a greater extent under stressful conditions (Armbruster & Reed, 2005; Crnokrak & Roff, 1999; Fox & Reed, 2011). Accordingly, the importance of considering inbreeding as a major threat to population persistence is increasing under rising frequencies of extreme environmental events associated with climate change.

Genetic problems of small and isolated populations can be mitigated by the introduction of genetically diverse individuals through restoring habitat connectivity and/or translocation of genetically diverse wild‐ or captive‐bred offspring (Frankham, 2015; Whiteley et al., 2015). The resultant augmented gene flow can increase population fitness, alleviate inbreeding and elevate the adaptive potential of rescued populations, thus increasing the probability of persistence in the face of environmental changes (Harrisson et al., 2014). Sourcing individuals for augmented gene flow from genetically diverse, recently diverged populations occupying similar environments often results in improved health and growth of inbred populations (i.e. genetic rescue) without negative fitness effects (outbreeding depression; Frankham et al., 2014). Despite the urgency of conservation interventions and the alluring simplicity and cost‐effectiveness of augmented gene flow, this approach is not yet a mainstream management tool and test cases on how best to perform it remain in demand (Bell et al., 2019; Fitzpatrick et al., 2023).

One species in which genetic management is deemed essential for preventing population extinctions is the Macquarie perch *Macquaria australasica*, an endemic south‐eastern Australian freshwater fish (Pavlova, Beheregaray, et al., 2017). From being widespread and common before the settlement of Australia by non‐indigenous people ~240 years ago, the Macquarie perch has suffered a strong population decline, range contraction and severe habitat fragmentation and alteration (Lintermans, 2023a; Trueman, 2011). With the continuing decline of its remaining populations, the Macquarie perch is listed as Endangered under the Environment Protection and Biodiversity Conservation Act and the IUCN Red List of Threatened Species (Lintermans et al., 2019). Genetic risk assessment found most remnant populations to be depleted of genetic diversity, with effective population sizes below the threshold needed to maintain long‐term adaptive capacity (*N_e_* = 1000), and in some populations below the threshold required to prevent inbreeding depression in a short‐term (*N_e_* = 100) (Pavlova, Beheregaray, et al., 2017). Consequently, translocations were recommended to increase genetic diversity and alleviate inbreeding. These recommendations were adopted in the National Recovery Plan for Macquarie perch (Commonwealth of Australia, 2018).

Contemporary methods for successful translocations of Macquarie perch are still under development (Lintermans et al., 2015; Lutz et al., 2021; Todd & Lintermans, 2015). Successful unintended gene pool mixing has occurred historically in several populations (Lutz et al., 2022; Pavlova, Beheregaray, et al., 2017), but mixing gene pools as a tool for genetic augmentation was first applied in 2010 during the restoration of the extinct Ovens River population through stocking and translocations using two sources (Yarra River and Lake Dartmouth) following habitat rehabilitation (Lutz et al., 2021). Genetic monitoring of the Ovens population showed that offspring of river fish (very genetically diverse) and offspring with parents from two populations had better survival after stocking compared to offspring with less genetically diverse, lake‐only ancestry. Moreover, translocated river fish contributed more to recruitment compared to lake fish, despite many more lake individuals having been translocated. While promising for beneficial genetic mixing, this work raises questions around the availability of riverine individuals for translocation and whether using lake individuals might be less effective than riverine individuals. The relative importance of different management strategies might be population‐specific, which could be assessed without halting management interventions by applying of an adaptive management (‘learning‐by‐doing’) framework. In the context of genetic management, this means that initial genetic augmentation trials are followed by intensive monitoring and analyses assessing their effectiveness, with results informing future actions.

Here, we present a test case of a genetically monitored attempt at genetic rescue of the Cotter River Macquarie perch population using a closely related and more genetically diverse lake population as a source, which aimed to aid planning and implementing future interventions. We also considered water flow regulation in this river as an additional management action, as highly variable water flows may impact breeding opportunities and juvenile growth and survival. During low‐flow conditions, reduced water depths in the Cotter River result in multiple natural in‐stream barriers precluding upstream movement of fish between pools (Broadhurst et al., 2016). Low flow can also create stressful environments that impede the growth of young fish, for example, through changes in water oxygenation and nutrients (Humphries et al., 2020; King et al., 2009), and reducing refuges from environmental extremes and predators. Because inbreeding is expected to have the strongest effect under stressful conditions (Crnokrak & Roff, 1999), growth rates of young fish in stressful years may depend on their individual level of inbreeding, even if inbreeding depression in years with favourable conditions is undetectable. Meanwhile, high flows can wash out eggs, reducing juvenile survival.

As a case study of adaptive management focussed on genetic rescue, we report on the initial implementation of augmented gene flow (71 individuals translocated over 3 years), assess its success through genetic monitoring and develop recommendations to facilitate genetic rescue. Using water flow, genetic and morphometric data, we address the following questions: (1) Do Cotter Macquarie perch experience inbreeding depression, and if so, are the negative effects of inbreeding more pronounced in more stressful years? (2) Did translocated individuals breed, interbreed with local fish and augment population genetic diversity of the Cotter River population? (3) Based on the observed outcomes, how many additional individuals need to be translocated to reduce the population inbreeding coefficient to 0.1 (a target recommended by Frankham et al., 2019) and from where should they be sourced? (4) Did water flow levels affect the number of breeding adults (*N*_b_), genetic diversity of offspring and juvenile dispersal? The answers will inform range‐wide genetic management of Macquarie perch, and contribute to a broader understanding of implementation of augmented gene flow and genetic management in regulated rivers. Additional questions arose from the untested perception that different subpopulations of resident Macquarie perch inhabit the Cotter River and downstream Cotter Reservoir (Broadhurst et al., 2019, 2020; Ebner & Lintermans, 2007; Lintermans et al., 2010), and we are able to showcase how such questions can be addressed by asking: (5) Does genetic diversity of the Cotter fish decline with distance from the reservoir? (6) Is there evidence of spatial genetic structure consistent with limited interbreeding of river and reservoir fish? Better understanding fish movement during breeding and juvenile dispersal and other processes structuring genetic diversity can further inform translocations and flow management in regulated rivers. Co‐design of management‐focused research such as we demonstrate here has substantial potential to close the gap between practitioners and researchers that has been of considerable concern (Taylor et al., 2017).

## 2 METHODS

### 2.1 Study area, water flow regulation and sampling sites

The Cotter River (north‐western Australian Capital Territory, ACT) is a perennial river of the Murrumbidgee catchment within the Murray‐Darling Basin. It runs through the traditional lands of the Ngunnawal Aboriginal Nation, providing a major water supply to the ACT region (Lintermans, 2012). The Cotter Dam (built in 1915) isolated the Cotter River and Murrumbidgee River Macquarie perch (Gubay in the Ngunnawal language) populations and resulted in the formation of a lake, Cotter Reservoir. Further, connectivity along the Cotter River became restricted by constructed road crossings (Appendix S1) and reduced river flows, regulated by upstream dams (Lintermans, 2002).

The upstream Bendora Dam (constructed in 1961), regulates water flows in the Cotter River (Appendix S3, Figure C1). The volumes of environmental flow released from Bendora Dam are based on river function requirements. A daily base flow is released of 75% of the 80th percentile of long‐term monthly flow (maximum of 3888 ML/month in September to a minimum 530 ML/month in March for a total of 21.1 GL per year) or the current inflow into Bendora, whichever is less. A flow of 150 ML/day for 3 days is released bimonthly (including October and December) for riffle maintenance; through the study, there were several periods where only inflow was provided, particularly in the 2018–19 season. Additionally, a pool maintenance flow of greater than 550 ML/day for 2 days is provided biannually when possible at irregular timing, unless it is provided from natural flow; during the study period pool flushes were not delivered. Flows change the passability of ~40 natural medium‐to‐large in‐stream barriers by Macquarie perch in the upstream direction (Appendix S1, Table A1). Under flows <32 ML/day, these barriers preclude the movement of adults to spawning sites, although stepping‐stone upstream movement across seasons may still occur (Broadhurst et al., 2016). Under moderate environmental flows of 150–204 ML/day, currently delivered every 2 months, half of the medium‐sized (0.3–0.5 m high) barriers become easier for fish to cross. The remaining medium‐size and all large (>0.5 m high) barriers become passable only under higher flows such as rare natural flood or dam‐overtopping conditions (Broadhurst et al., 2016). Although such conditions are likely to occur during pool flushes, their timing (between July and October) does not often coincide with Macquarie perch movement to spawning sites (October–November). All flow conditions appear sufficient for downstream movement by fish of all ages.

**FIGURE 1:**
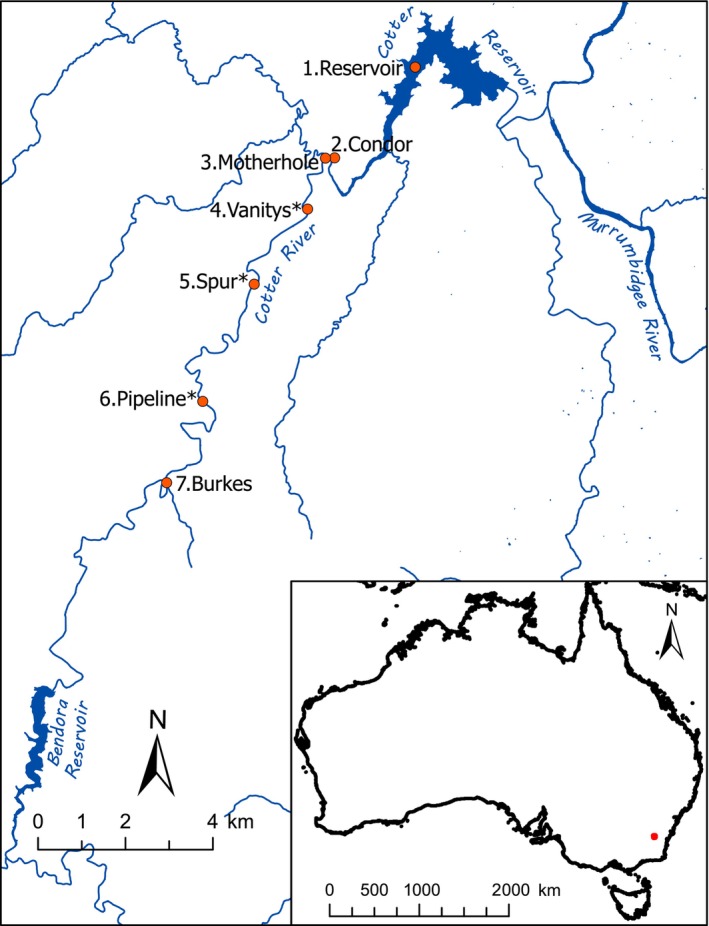
Sampling locations along the Cotter River (1.Reservoir is the most downstream site). Translocated Cataract Dam fish were released at three sites marked with asterisks: Pipeline (*N* = 23), Spur (*N* = 24) and Vanitys (*N* = 24). The inset map shows Australia, with a red dot including the area of the main map.

We focused on seven sites in the ~18 km‐long lower reach of the Cotter River downstream of Bendora Dam, spanning the current range of the Cotter Macquarie perch population: 1. Reservoir (Cotter Reservoir, ~2.3 km long × 0.5 km wide lake constructed in 1915, enlarged to ~6.5 km long since 2014; the most downstream site), 2. Condor (Condor Confluence), 3. Motherhole, 4. Vanitys (Vanitys Crossing), 5. Spur (Spur Hole), 6. Pipeline (Pipeline Rd Crossing) and 7. Burkes (Burkes Creek Crossing, the most upstream site; Figure 1). Macquarie perch does not occur in or above Bendora Reservoir.

### 2.2 Macquarie perch breeding biology

Macquarie perch have a lifespan of up to 26–30 years (Todd & Lintermans, 2015). During breeding, they lay eggs in runs and riffles where flowing water keeps eggs oxygenated and clear of sediment (Broadhurst et al., 2019; Tonkin et al., 2010). Spawning occurs primarily in November, with eggs hatching by the beginning of December (Broadhurst et al., 2019; Lintermans, 2023a). Thus, October–November flows are important for the movement of breeders, while December–January conditions are crucial for hatching, survival and initial growth of larvae. High water flow during spawning can wash out spawning sites and damage larvae thereby reducing recruitment (Tonkin, Kearns, et al., 2017).

Based on restricted spawning movements reported for reservoir‐bound Macquarie perch, which must enter flowing water to spawn (Tonkin et al., 2009, 2010), it is inferred that resident Cotter Reservoir fish spawn predominantly in the nearest available spawning sites (Condor and Motherhole), with Vanitys and more‐upstream spawning sites accessed only by resident river fish (Broadhurst et al., 2016, 2019). Such use of distinct breeding sites by river and reservoir Macquarie perch is expected to reduce gene flow between river and reservoir subpopulations and exacerbate genetic drift, elevating local extinction risk. High‐resolution genetic data can shed light on fish movement along rivers (Coleman et al., 2018; Grummer et al., 2019). Restricted interbreeding between river and reservoir fish is expected to generate spatial genetic structure and result in low genetic diversity at sites further from the Cotter Reservoir.

### 2.3 Genetic management: Translocations, monitoring and genetic sampling

The Cotter River population of Macquarie perch has extremely low genetic diversity and was predicted to experience inbreeding depression based on low effective population size (Farrington et al., 2014; Pavlova, Beheregaray, et al., 2017). Accordingly, translocations were strongly recommended to augment genetic diversity and evolutionary potential to prevent extinction through inbreeding depression (Pavlova, Beheregaray, et al., 2017). Following earlier recommendations, the Cataract Dam Macquarie perch population (established in ~1915 from Murrumbidgee stock; Legislative Assembly of New South Wales, 1916) was selected as the source of individuals for initial translocations into the Cotter River, based on high genetic diversity and low risk of causing harmful fitness effects due to historical connectivity of the Cotter and Murrumbidgee populations (Pavlova, Beheregaray, et al., 2017; Pavlova, Gan, et al., 2017). Translocations were conducted for three consecutive years (2017–2019) with 12–31 fish moved per year (71 total; Appendix S1, Table A2). Whereas in the Cotter Reservoir males mature at 140–150 mm and females at 300 mm (Lintermans, 2023a), Cataract Dam individuals mature at a smaller size (90 and 95 mm, respectively) and younger age (2.5 years) due to limited resource availability and strong predation pressure on large individuals by locally abundant Murray cod (Stocks et al., 2020). Translocated individuals were 123 to 205 mm (mean = 155, SD = 17.5) and hence inferred to be mature. They were released to three sites—Pipeline, Spur and Vanitys (Figure 1)—following quarantine in captivity for 30 days and blood testing to prevent the spread of epizootic haematopoietic necrosis virus (Becker et al., 2013).

Monitoring and sampling of the Cotter River Macquarie perch population occurred predominantly in February and March from 2002 to 2021 across seven sites (Figure 1) (Lintermans et al., 2013). Fish captured mainly using fyke nets or backpack electrofishing were measured and fin‐clipped to obtain tissue samples before being released (methods in Lintermans, 2016). Each December from 2016 to 2020, a small number of larvae were lethally collected during post‐translocation snorkelling surveys. Overall, we used 169 pre‐translocation individual samples from 2002–2016, 447 post‐translocation samples from 2017 to 2021 and 71 samples of translocated fish (total *N* = 687; Appendix S1, Table A2). Tissue samples were stored in ethanol at room temperature and/or at −20°C.

### 2.4 Identifying cohorts of fish born in the same year

For individuals collected from 2016 onwards, total length was used to estimate age and birth year, using breaks in the empirical distribution of lengths (Appendix S1, Figure A1). These age groups and capture dates were used to assign individuals to five birth year cohorts, 2016 to 2020 (Appendix S1, Figure A2), with assignments corroborated by members of most full‐sibling families belonging to the same cohort (see Results). Fish sampled before 2016 (in 2002–2013) were analysed together in their own category (referred to as ‘multi‐age group’) because length measurements were missing for many individuals. The range of recorded lengths for the multi‐age group (156–332 mm) corresponded to sub‐adult to mature individuals.

### 2.5 DNA extraction, genotyping and filtering

DNA was extracted from the fin clips using a DNeasy Blood and Tissue Kit (Qiagen) and genotyped commercially using Diversity Arrays Technology Pty Ltd (DArT). Reduced‐representation sequencing libraries were prepared using DartSeqTM protocols (Kilian et al., 2012) and sequenced using Illumina technology. Single Nucleotide Polymorphism (SNP) calling followed by Nguyen et al. (2018).

Genotypes were imported to R v4.0.5 (R Core Team, 2021) with RStudio v1.2.5042 (RStudio Team, 2020) using *dartR v2.0.4* (Mijangos et al., 2022). Genotypes were filtered by removing individuals with >30% of missing data, loci with reproducibility <95%, loci missing in >20% of individuals and monomorphic loci. We further removed loci likely to represent incorrectly merged loci (78 loci with heterozygosity significantly (*p* < 0.05) larger than the maximum expected value of 0.5 in either Cotter or Cataract populations) using *R* function *filter.excess.het* (Robledo‐Ruiz et al., 2023). We did not apply the function to the Cotter‐only dataset where multi‐age group/cohorts represented populations because excess of heterozygosity could result from admixture between previously isolated populations (Wahlund‐breaking), a suspected outcome of the 2013 Cotter Reservoir expansion. Instead, for the Cotter‐only dataset we removed loci detected as above using *dartR* function *gl.drop.loc*. One random SNP per DArT‐tag was retained. Sexing data were not available, but none of the DArT loci were expected to be strongly sex‐linked because the species has no defined sex chromosomes (Pavlova et al., 2022). The final complete Cotter + Cataract dataset comprised 5116 biallelic SNPs scored for 678 individuals (610 Cotter and 68 Cataract Dam; Table 1; Appendix S1, Table A3), and a Cotter‐only dataset (i.e. after removing Cotter × Cataract crosses, see Section 3) comprised 4338 SNPs scored for 608 individuals with Cotter‐only ancestry. Smaller subsets of data were created for multi‐age groups and each cohort using the same filtering criteria.

**TABLE 1:** Number of analysed genotypes for the multi‐age group (2002–2013) and each cohort across seven Cotter River locations, numbered from the most downstream (1.Reservoir) to the most upstream site (7.Burke) and Cataract Dam individuals translocated from 2017 to 2019.

Sampling site	Cohorts of cotter fish						Cotter total
	2002–2013	2016	2017	2018	2019	2020	
1.Reservoir	30				13		43
2.Condor		10	16	18	1		45
3.Motherhole		10	23	24	6	2	65
4.Vanitys	32	10	45	33	21*	26	167
5.Spur	22	34	9	78	64*	16	223
6.Pipeline	1	13	12	12	14		52
7.Burkes		6		9			15
Cotter total	85	83	105	174	119	44	610

**Sampling site**	**Translocation years (from Cataract Dam)**						**Cataract**
		2017	2018	2019			Total
Cataract Dam			28	28	12		68
Total number of genotypes	85	83	132	206	131	44	678

*Note*: Asterisks indicate samples which include admixed individuals (one each). The distribution of age categories for each cohort is in Appendix S1, Figure A2.

For parentage/sibship analyses in *Colony2* (below), we opted for more stringent filtering: we removed individuals with >20% of missing data, loci with reproducibility <0.99 and SNPs with the greatest information content per tag were retained (*dartR* script *gl.filter.secondaries*, method ‘best’); the remaining filtering steps were as above. The complete Cotter + Cataract dataset for *Colony2* comprised 3149 SNPs scored for 675 individuals (610 Cotter and 65 Cataract Dam) and the Cotter‐only dataset was 2590 SNPs scored for 608 non‐admixed Cotter individuals. Smaller per‐cohort *Colony2* datasets were also created using the above filtering criteria. R function *gl2colony* (Robledo‐Ruiz et al., 2023) was used to create *Colony2* input files within R.

### 2.6 Genetic structure

Genetic structure was assessed using Principal Coordinate Analysis (PCoA) implemented by *dartR* function *gl*.*pcoa*. First, the complete Cotter + Cataract dataset was used to test whether the translocated Cataract Dam fish bred in the Cotter River, and whether they interbred with local fish. Based on strong differentiation at microsatellite loci (Pavlova, Beheregaray, et al., 2017), Cataract and Cotter samples were expected to be clearly differentiated by PC1 values, with cross‐population offspring having intermediate values. Second, we tested the Cotter‐only dataset for geographic structure consistent with reservoir and river subpopulations having limited gene flow between them. For that, we conducted a PCoA for the Cotter‐only dataset, and AMOVA (Analysis of Molecular Variance; Excoffier et al., 1992), implemented by *dartR* function *gl.amova*, to estimate molecular variance explained by sampling site. Third, we explored whether geographic structure changed over time, by conducting AMOVA on six time/birth cohorts. We considered that after the 2013 Cotter Reservoir enlargement and high flows of 2016 that inundated the in‐stream barriers, reservoir fish were able to use the more upstream Condor spawning site for breeding and this may have removed previous differentiation between the river and reservoir populations.

### 2.7 Identity, parentage and sibship analyses

*Colony2* v 2.0.6.5 (Jones & Wang, 2010) was used to perform joint identity, parentage and sibship analyses. Parentage assignments from the complete Cotter + Cataract dataset were used to identify translocated parents of Cotter offspring, and Cotter parents of admixed fish. Instances of multiple copies of the same genotype (i.e. the same individual sampled twice or identical siblings) and full sibship assignments of Cotter‐only analyses were used to infer dispersal patterns.

After multiple preliminary runs, final analyses were performed assuming codominant markers, allelic dropout rate of 0.01, other error rate of 0.001, dioecious, diploid, polygamous individuals and inbreeding. The analysis employed the most accurate full‐likelihood method using high precision (default), and medium run‐length while updating allele frequencies. Full sibship scaling was implemented with no prior sibship assumed. For parentage/sibship analyses, all Cotter individuals were included as potential offspring, all translocated Cataract Dam fish (when included) and Cotter River fish born up to 2018 inclusive were used as potential parents. Each potential parent was included as a candidate mother and father because sex was unknown. The probability of having a parent sampled was set to 0.2. Five replicate runs were performed by setting five different seeds chosen at random. Parent‐offspring and full‐sib families were accepted if they were found by at least three replicates.

### 2.8 Juvenile dispersal

We inferred dispersal distances using the geographic distance between full siblings. Data for 66 families (2 to 32 full siblings per family) born from 2016 onwards was used to test whether mean daily water flow averaged per birth year and/or family size explains the maximum geographic distance between full siblings of each family. We used flow data (mean daily discharge, in megaliters per day) measured at Vanitys Crossing in the Cotter River from 2016 to 2020 (ALS Global and Icon Water gauge data available at http://www.bom.gov.au/waterdata/; Appendix S3). A linear model with two fixed predictors—flow and family size—was fitted to the maximum distance between siblings, using the *lm* function in R package *stats*. Family size was included because larger families are more likely to capture rare long‐distance movements. The direction of movement (upstream/downstream) for each full‐sib family of five or more members was inferred based on the location of a genotype sampled later compared to the location of its sibling sampled earlier. In cases where some family members moved downstream and some upstream, or where the direction of movement could not be determined, direction was designated as ‘unclear’.

### 2.9 Effective population sizes

We used two methods to estimate the current effective population size, *N*_e_, from the multi‐age group and each of the five cohorts. Analyses were run on all individuals per cohort, and, to improve comparability across cohorts, separately on individuals from two sites where all cohorts were well‐sampled (*N* > 8 per cohort; Spur and Vanitys; Table 1). First, we estimated *N*_e_ with the linkage disequilibrium (LD) method LD*N*_e_ (Waples & Do, 2008) implemented in *NeEstimator* version 2.0 (Do et al., 2014), using *dartR* wrapper *gl.LDNe*, assuming random mating and ignoring loci with minor allele frequency < 0.01 (*P*_crit_ = 0.01). The less stringently filtered SNP dataset was used, because LD*N*_e_ can handle arbitrary amounts of missing data (Waples & Do, 2008). As the method assumes discrete generations and closed populations (i.e. no gene flow), estimates were performed without the admixed individuals identified in the PCA analysis, however, we also ran analyses including these individuals for comparison. Confidence intervals (95%) were calculated using the jackknife‐across‐samples method. To ensure the robustness of our estimates, we also estimated *N*_e_ using the sibship assignment (SA) method in *Colony2*, using the same data as for LD*N*_e_, and also using the more stringently filtered data used for parentage analysis. SA calculates maximum likelihoods using the frequencies of full‐sib and half‐sib dyads while assuming discrete generations and closed populations; random mating was further assumed. For these per‐cohort analyses, individuals were included as offspring only. As above, we ran these analyses for the 2019 cohort with and without admixed individuals. A single run per dataset was used.

Understanding the effective number of breeders *N*_b_ is important for evaluating inbreeding effective size relevant to the threshold of *N*_e_ > 100 required to prevent inbreeding depression and limit loss of fitness over five generations to <10% (Frankham et al., 2019). Due to age structure and generation overlap, the effective population size of an iteroparous species estimated by LD*N*_e_ from the same cohort (${\hat{N}}_{b}$) reflects a harmonic mean of the effective number of breeders in the parental generation (*N*_b_) and the effective size per generation (*N*_e_), but can be adjusted to reflect true *N*_b_ following recommendations of Waples et al. (2014), using adult life span (AL) and age at maturity (*α*), as $N_{b_{\mathrm{adj}}}$ = ${\hat{N}}_{b}$/(1.03–0.245 × log(AL/*α*)). For Macquarie perch with an adult life span of 23 years and age at maturity of three years, the effective number of breeders is ${\hat{N}}_{b_{\mathrm{adj}}}$ = ${\hat{N}}_{b}$/0.8133 (Pavlova, Beheregaray, et al., 2017). This $N_{b_{\mathrm{adj}}}$ could further be used to estimate effective size per‐generation $N_{e_{\mathrm{adj}}}$ using the formula of Waples et al. (2013): *N*_b_/*N*_e_ = 0.485 + 0.758 × log (AL/α). For Macquarie perch, *N*_b_/*N*_e_ = 1.156, so $N_{e_{\mathrm{adj}}}$ = $N_{b_{\mathrm{adj}}}$/1.156. Per‐generation *N*_e_ is relevant to the global threshold of *N*_e_ > 1000 required to retain population adaptive potential through the maintenance of mutation‐drift equilibrium (Frankham et al., 2019). LD*N*_e_ ${\hat{N}}_{e}$ estimated from a mixed‐age adult sample (such as Macquarie perch time cohort 2002–2013) also approximates per‐generation *N*_e_ (albeit consistently downwardly biased), with low bias expected when *N*_b_/*N*_e_ is close to one, and when the number of cohorts in the sample is close to the generation length (seven years for Macquarie perch; Pavlova, Beheregaray, et al., 2017), but the above approach of estimating $N_{e_{\mathrm{adj}}}$ based on individual cohorts is considered the most reliable (Waples et al., 2014). We note that Waples et al. (2014) used LD*N*_e_ with *P*_crit_ = 0.05, and did not model smaller critical values (such as *P*_crit_ = 0.01 used here) which may increase precision at the cost of some upward bias in the estimates.

### 2.10 Individual heterozygosity and genetic diversity

Individual heterozygosity was estimated as the proportion of heterozygous loci per individual (PHt), calculated using *dartR* function *gl.report.heterozygosity*, method = ‘ind’. First, we used the complete Cotter + Cataract dataset to calculate average PHt values for the three sets of Cataract individuals (translocated in 2017, 2018 and 2019), and the Cotter multi‐age group and five cohorts. These data were used to confirm higher diversity in Cataract than Cotter individuals and calculate the number of translocated fish needed to reduce inbreeding. Individual estimates informed whether admixed individuals were more genetically diverse than either Cotter or Cataract populations, which would indicate that attempted genetic rescue from Cataract Dam is bringing novel or lost variation to the Cotter.

Next, we calculated individual PHt for the Cotter‐only dataset (excluding admixed individuals), and used these values in four separate linear models (LM), to test for the following relationships: (LM1a) Does PHt decrease over time? A decrease in PHt across cohorts would be expected under strong genetic drift. (LM1b) Does PHt differ across cohorts (analysed as categories)? We compared pairs of cohorts using a post hoc Tukey Honest Significant Differences (HSD) test implemented in *TukeyHSD* function of *stats* package in R, following an Analysis of Variance (ANOVA) fitted with *aov*. (LM2) Does PHt decrease with distance from Cotter Reservoir? A decrease in PHt with distance would be expected if fewer breeders are reaching upstream sites for breeding. (LM3) Do older individuals have higher PHt? Individuals with higher heterozygosity are expected to live longer under inbreeding depression. (LM4) Are old individuals found further upstream or close to Cotter reservoir? A negative relationship between age and distance to Cotter reservoir would indicate the prevalence of downstream dispersal with age and a positive relationship prevalence of active upstream dispersal. All models were run on individuals born 2016 onwards (e.g. data for five cohorts).

### 2.11 Inbreeding and inbreeding depression

We evaluated whether the level of individual inbreeding corresponds to slower growth in the first 2 years of individuals' lives, and if so, whether inbreeding depression is stronger in years with more stressful environmental conditions. The assumption of faster growth corresponding to higher fitness is common in studies of ectothermic aquatic vertebrates and supported by reports of negative relationships between inbreeding and growth in many fish species (Kincaid, 1983; Thrower & Hard, 2009). We used PHt to approximate the level of inbreeding, where lower values correspond to stronger inbreeding (Harrisson et al., 2019). We focussed on 438 juveniles of Cotter‐only ancestry (identified by PCA on combined data) of three age groups: young‐of‐year (YOY) larvae, older YOY and 1–2YO juveniles (Appendix S1, Figure A1). Growth of Macquarie perch with age was approximated by the standard Gompertz growth model (Tonkin, Kearns, et al., 2017) (Appendix S2), and residuals from this model were used as the response in the inbreeding depression model. These growth residuals indicate whether the individual is longer or shorter than the average for its age. Because juvenile growth depends on the availability of resources, we first fitted a linear model (LM5) testing whether cohort (analysed as category) predicts growth residuals, and compared residuals between pairs of cohorts using Tukey's test. To test for inbreeding depression, we fitted LM6, with PHt as a single predictor of growth residuals, followed by a linear mixed model LMM6, using the *lme* function of R package *nlme*, to control for random differences in environmental effects at birth. In LMM6, birth year was used as a single random effect, PHt was used as a single fixed predictor. Models were compared using ANOVA to check whether adding the random effect significantly improved the model. Finally, to test whether one or more cohort drives any negative effect of inbreeding on growth, we fitted LM6 to data for each cohort. Attempts to implement the alternative approach of fitting the model with an interaction term PHt*cohort were unsuccessful, apparently due to over‐parameterization (details in Data S1).

To ensure impartiality of result interpretation, the experts (ML and MB) were asked to predict before seeing the results which years, from 2016 to 2020, they believe to have been stressful for juvenile Macquarie perch. Low water flow presents unfavourable conditions for young fish and has been associated with major fish kills (Koehn, 2022); the years with the lowest flows in the Cotter River were 2018 and 2019 (Table 2; Appendix S3, Figure C1). Thus, the cohort of fish born in 2018 (sampled as larvae in 2018, YOY in 2019 and 1‐2YO in 2020) was expected to have been growing under comparatively stressful conditions. This prediction was also supported by the observation of extremely low abundance of young‐of‐year Macquarie perch (<80 mm total length) across four of five surveyed sites in the Cotter River in 2018 (Broadhurst et al., 2020). While flow was also low in 2019, it was reasonably high the year after, and abundance of young‐of‐year fish observed across most Cotter sites in 2019 was relatively high. Although 2016 had a very high flow, it could also have been predicted to be relatively stressful for young‐of‐year fish, based on their low abundance across all five surveyed Cotter sites, as might have been expected from eggs being washed out by high flow.

**TABLE 2:** Average flow values and estimates of population parameters for the multi‐age group (2002–2013) and five cohorts.

Parameters	Cohorts					
	2002–2013	2016	2017	2018	2019	2020
Mean daily flow (Ml/day)						
Birth year average	NA	222	97	48	52	130
Growth residuals, Mean (SD)						
All sites; with admixed	NA	−3.69 (7.81)	−5.26 (8.42)	5.22 (7.71)	1.78 (7.30)	−3.42 (4.95)
Maximum dispersal distance (km) between siblings, Max (Mean of max distance across families)						
	NA	9.4 (2.6)	4.5 (1.0)	4.5 (1.6)	2.9 (1.4)	4.5 (1.7)
Heterozygosity, Mean (SD)						
All sites; no admixed	0.102 (0.011)	0.102 (0.009)	0.101 (0.011)	0.094 (0.013)	0.098 (0.011)	0.099 (0.011)
All sites; with admixed					0.100 (0.019)	
LD*N*_e_ ${\hat{N}}_{e}$ (multi‐age group 2002–2013) or per‐cohort ${\hat{N}}_{b}$ (low‐high JackKnife on samples)						
All sites; no admixed	54 (45–69)	53 (44–63)	26 (21–32)	29 (26–33)	19 (16–23)	44 (35–55)
All sites; with admixed					18 (14–23)	
Spur+Vanitys; no admixed	29 (23–37)	65 (55–92)	17 (13–22)	16 (14–19)	11 (9–12)	44 (34–53)
Spur + Vanitys; with admixed					3 (1–18)	
Effective number of breeders in one reproductive cycle $N_{b_{\mathrm{adj}}}$ = ${\hat{N}}_{b}$/0.8133						
All sites; no admixed	NA	65 (53–77)	32 (26–39)	35 (31–40)	23 (19–28)	54 (43–67)
Spur + Vanitys; no admixed	NA	80 (67–113)	21 (16–27)	20 (17–23)	13 (11–15)	54 (42–64)
Effective population size per‐generation $N_{e_{\mathrm{adj}}}$ = $N_{b_{\mathrm{adj}}}$/1.156						
All sites; no admixed	NA	56 (46–67)	28 (23–34)	30 (27–35)	20 (16–24)	46 (37–58)
Spur + Vanitys; no admixed	NA	69 (58–98)	18 (14–24)	17 (15–20)	11 (9–13)	47 (36–56)
*Colony2* per‐cohort *N*_b_ (lower‐upper 95% confidence interval)						
All sites; no admixed	46 (31–71)	36 (23–59)	23 (14–43)	20 (12–42)	16 (9–34)	15 (9–34)
All sites; with admixed					17 (10–34)	
Spur + Vanitys; no admixed	25 (15–46)	21 (12–42)	12 (6–28)	10 (5–24)	9 (5–24)	13 (7–30)
Spur + Vanitys; with admixed					9 (5–24)	
Number of Cataract Dam fish released			31	28	12	

*Note*: Mean heterozygosity was calculated from individual PHt (proportion of heterozygous sites per individual in the dataset that included all Cotter genotypes) averaged per cohort either excluding (no admixed) or including (with admixed) two Cotter × Cataract offspring for the 2019 cohort. Parameters were calculated using all sampling locations (all sites) or only two sites where all cohorts were well represented (Spur + Vanitys). *N*_e_ or *N*_b_ are estimated by LD*N*_e_ and *Colony2* using the same less stringently filtered dataset. LD*N*_e_ estimates of ${\hat{N}}_{b}$ from cohorts can be adjusted to reflect the true effective number of breeders in one reproductive cycle *N*_b_ using $N_{b_{\mathrm{adj}}}$ = ${\hat{N}}_{b}$/0.8133, and effective population size per generation using $N_{e_{\mathrm{adj}}}$ = $N_{b_{\mathrm{adj}}}$/1.156 (see Methods). LD*N*_e_ estimate ${\hat{N}}_{e}$ from the multi‐age group (2002–2013) reflects downwardly biased per‐generation *N*_e_.

Abbreviation: NA, not applicable.

### 2.12 Estimating the number of effective migrants needed to reduce population inbreeding to *F* = 0.1

Given that at population‐level inbreeding of *F* = 0.2 inbreeding depression is often observed in wild populations (Frankham, 1995), conservation management should aim to reduce it, with *F ≤* 0.1 often used as an acceptable goal. We estimated the number of non‐inbred, unrelated migrants needed to reduce the population‐level inbreeding in the Cotter River to *F =* 0.1 following Frankham et al. (2017). First, we calculated population‐level inbreeding of the Cotter Macquarie perch (*F*_Cotter_) by comparing its heterozygosity to that of the Cataract Dam population, assumed to be outbred (*F*_Cataract Dam_ = 0), using *F*_Cotter_ = 1 − (PHt_Cotter_/PHt_Cataract Dam_). Next, we used the equation 12.2 of Frankham et al. (2017) to calculate the proportion of the augmented population derived from the unrelated migrants (*f*_Cataract Dam_), using *f*_Cataract Dam_ = 1‐sqrt(*F*_augmented_/*F*_Cotter_), using the target of 0.1 for *F*_augmented_. Finally, we used *f*_Cataract Dam_ to estimate the number of successful breeding migrants needed to reduce inbreeding (*N*_m_), assuming that these migrants will increase the current pool of Cotter breeders (*N*_b_, estimated as LD*N*_e_ $N_{b_{\mathrm{adj}}}$ averaged across cohorts). Given that *f*_Cataract Dam_ = *N*_m_/(*N*_b_ + *N*_m_), solving for *N*_m_ results in the following equation: *N*_m_ = *f*_Cataract Dam_ × *N*_b_/(1–*f*_Cataract Dam_).

## 3 RESULTS

### 3.1 Population structure

**FIGURE 2:**
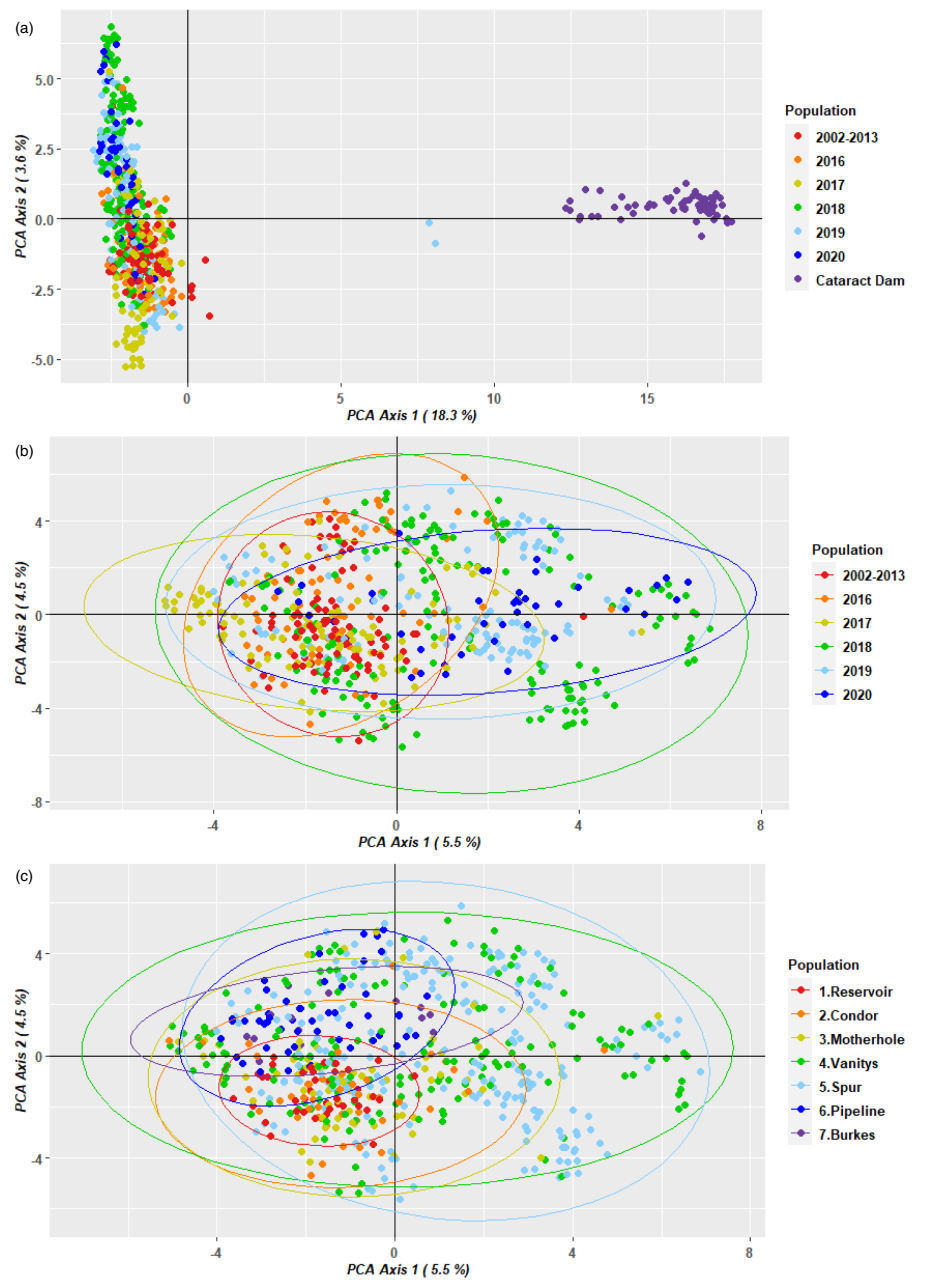
Principal Component Analysis of (a) Cotter + Cataract and (b, c) Cotter‐only dataset (admixed individuals excluded). PC1 on plot (a) shows a clear separation of the Cotter individuals (left cloud) and Cataract Dam individuals (right cloud); two individuals with intermediate values of PC1 are admixed Cotter × Cataract crosses born in 2019 (MP_L172 sampled at Vanitys in 2020 and MP_L204 sampled at Spur in March 2021). Cotter points on (a) and (b) coloured by the multi‐age group/cohorts, on (c) by sampling sites, numbered from downstream (red) to upstream (purple; bottom). Ellipses encapsulate 95% of points for each population.

Cotter and Cataract Dam fish were clearly differentiated on the first axis of the Principal Coordinate Analysis (PCoA) on the complete Cotter + Cataract dataset (Cotter PC1 < 1, Cataract PC1 > 12; Figure 2a, Appendix S4; PC1 explained 18.4% of the total variance). Two Cotter River individuals born in 2019 had intermediate values (7.9 and 8.1: MP_L172 sampled as YOY at Vanitys in 2020 and MP_L204 sampled as 2–3YO at Spur Hole in 2021); these were inferred to be admixed offspring of one Cotter and one Cataract Dam parent. This represents 0.45% of a total of 446 sampled Cotter fish born after the onset of translocations.

PCoA analysis of the Cotter‐only dataset showed no distinct clusters of individuals (Figure 2b,c). More recent cohorts (2018–2020; Figure 2b) and individuals occurring in geographically intermediate sites (Vanitys and Spur; Figure 2c) tended to vary more along PC1 compared to other cohorts and sites, indicating the presence of genotype combinations absent in older samples (2002–2013) and more downstream (Motherhole, Condor, Reservoir) or upstream sited (Pipeline, Burkes). PC2 tended to separate the most downstream and most upstream sites in the Cotter River, but with considerable overlap. AMOVA showed that cohorts explained 8.1% of all genetic variance, and sites 7.7% (*p* < 0.001). Separate PCoA analyses of the multi‐age group and cohorts showed that genetic structure across the Cotter River sampling sites was never strong, and varied across years (Appendix S4, Figure D1). Uneven sampling of sites across cohorts (Table 1) and the presence of family structure (see below) precluded further interpretation.

### 3.2 Parentage, identity and sibship analyses

Parentage analysis of the Cotter + Cataract dataset revealed that the two admixed individuals were offspring of the same Cataract Dam individual (MP_CDM93), which was translocated in 2019. The second parents of these offspring were inferred to be different, unsampled Cotter individuals. Thus, successful mating of at least one translocated Cataract Dam individual in the year of translocation with at least two Cotter Dam partners was inferred.

Identity analysis of the Cotter‐only dataset detected that eight individuals were sampled two or three times each (Appendix S5, Table E1). One of them was inferred to have dispersed 6 km downstream from Burkes to Pipeline between 2019 (when it was YOY) and 2020 (when it was 1–2YO). Three individuals were recaptured at the same location 1 to 2 years apart, suggesting a degree of site fidelity: one was sampled as 2–3YO and >4YO at Spur, one as YOY and 1–2YO at Spur and one as 1–2YO and 3–4YO at Vanitys. Four individuals (or pairs of identical twins) were sampled at the same site within a short time span (up to a week).

Across five replicate runs of the Cotter‐only dataset, three or more runs found 76 full‐sib families with two to 32 members each (mean 5.3 per family; Appendix S5, Tables E2 and E3; four families either included or comprised identical genotypes). A total of 402 individuals had full siblings in the dataset, half within the 14 largest families of 8 or more full‐sibs. The majority (95%) of families comprised members assigned to the same birth cohort, indicating that most full‐sib assignments were reliable. Two families had one sibling inferred to be born a year before or after the inferred birth year of the remaining full siblings, suggesting that their parents reproduced with each other in more than 1 year, or some of the offspring were smaller or larger than expected for their age. Two family assignments (three and two individuals) combined siblings from cohorts more than one year apart, and thus may represent other family relationships (e.g. parent‐offspring), unless their parents reproduced together more than once.

### 3.3 Juvenile dispersal

Dispersal was detected in some families but not others. Of 66 families with two or more full sibs born 2016 onwards, 45% comprised full‐sibs all sampled at the same site, 41% included full‐sibs sampled up to 3 km apart, 12% had full‐sibs found 3–5 km apart and one family of full‐sibs born in 2016—the year of highest flow—was sampled across sites 9.7 km apart, supporting the expectation of greater dispersal in high‐flow years (Appendix S5). Juveniles of any age appeared to have engaged in dispersal, as some families had siblings sampled as larvae at different sites (downstream‐only movement was presumed for larvae).

The maximum dispersal distance varied among individual families and increased with flow. A linear model fitting water flow averaged per birth year and family size as predictors of maximum distance between sampled full siblings significantly explained 11.5% of the variance in maximum distance (*p* < 0.01): larger families (*p* < 0.01) and higher flow levels (*p* < 0.05) corresponded to larger maximum distances between siblings (Table 2, Appendix S5, Tables E2 and E3).

The direction of movement varied across individual families, but was stronger in the downstream than upstream direction. Of 25 families of five or more full sibs born 2016 onwards (Appendix S5, Table E2), 24% of families did not disperse, in 28% some members dispersed downstream and 12% some members dispersed in an upstream direction; for 36% of families movement occurred but the direction was not possible to infer based on the geographic distribution of samples and sampling times of the siblings (including 8% of families where both downstream and upstream movement was possible). With rare exceptions (such as family 10 born in 2016), families occurring in the most upstream sites, Pipeline and Burkes, were not sampled elsewhere, suggesting their isolation in low‐flow years. However, high connectivity across the Cotter River in high‐flow years was evidenced by siblings of family 10 being sampled across five sampling sites from Pipeline to Condor.

### 3.4 Effective population sizes

**FIGURE 3:**
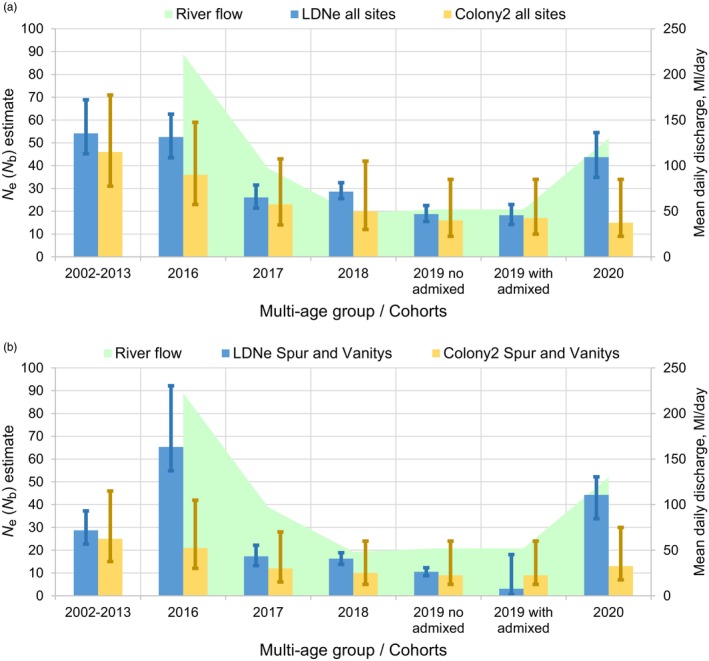
Estimates of short‐term effective population size for each multi‐age group/cohort by LD*N*_e_ (blue bars; *P*_crit_ = 0.01; errors are JackKnife on sample) or *Colony2* (yellow bars; errors are 95% Confidence Intervals). (a) Estimates include all sampling sites, (b) Only two sites sampled across all cohorts (Spur and Vanitys). The green background, and right *y* axis, indicate the mean daily water flow averaged per year (in megaliters per day).

When admixed individuals were excluded, LD*N*_e_ estimates of numbers of breeders (${\hat{N}}_{b}$) in five cohorts estimated over all sites ranged from 19 to 53 (when estimated over two common sites, Spur and Vanitys, from 11 to 65), were lowest for the three cohorts born in years of low flow (2017–2019) and highest for the cohort born in the high‐flow year 2016 (Table 2; Figure 3). When ${\hat{N}}_{b}$ were corrected for the biases due to iteroparity, age structure and overlapping generations, the number of breeders per reproductive cycle $N_{b_{\mathrm{adj}}}$ over all sites ranged from 23 to 65 (over the two common sites 13–80), and effective population size per‐generation $N_{e_{\mathrm{adj}}}$ over all sites ranged from 20 to 56 (over the two common sites 11–69). Per‐generation *N*_e_ estimated by LD*N*_e_ for the 2002–2013 multi‐age group was consistent with these adjusted estimates (${\hat{N}}_{e}$ = 54 over all sites, ${\hat{N}}_{e}$ = 21 over the two common sites). Including the two admixed individuals in the 2019 cohort resulted in a slightly lower estimate over all sites (${\hat{N}}_{b}$ 18 vs. 19), and a much lower estimate over the two common sites (${\hat{N}}_{b}$ 3 vs. 11).

The sibship‐assignment method implemented in *Colony2* yielded consistently lower *N*_b_ estimates than did LD*N*_e_ (15–36 breeding adults across five cohorts at all sites, 9–21 for the two common sites; Table 2), but showed a similar trend across cohorts, with the highest estimates corresponding to cohorts born in the high‐flow year (Figure 3). Including admixed individuals in the 2019 cohort resulted in similar estimates (over all sites 17 vs. 16, over the two common sites 9 vs. 9). *N*_e_ estimates from *Colony2* using the more stringently filtered dataset were similar or slightly smaller (Appendix S7).

The number of breeding adults in 2016–2020 (i.e. per‐cohort *N*_e_ estimated by LD*N*_e_ and *Colony2* for all data and just common sites) was correlated with daily flow averaged per year of breeding, significantly so for three out of four tests (LD*N*_e_ all sites: *R* ^2^ = 0.78, *p* < 0.05; LD*N*_e_ Spur and Vanitys: *R* ^2^ = 0.90, *p* < 0.01, *Colony2* all sites: not significant (*p* > 0.05), *Colony2* Spur and Vanitys: *R* ^2^ = 0.95, *p* < 0.01; Appendix S7).

### 3.5 Individual heterozygosity and genetic diversity

Average heterozygosity (approximated as a proportion of heterozygous loci per individual, PHt estimated from the complete Cotter + Cataract dataset) differed significantly between Cataract, Cotter and admixed fish (*p* < 0.001 for ANOVA and all comparisons of the post hoc Tukey's HSD test). Heterozygosity was higher for each of the three sets of Cataract Dam fish (translocated in 2017, 2018 and 2019: range 0.133–0.158) than the Cotter multi‐age group or any of the Cotter cohorts (range from 0.084 for 2018 to 0.091 for 2016; Appendix S6, Table F1, Figure F1). The two admixed individuals from the 2019 cohort had heterozygosity >2× higher than the most diverse Cotter individual and >1.2× higher than the most diverse Cataract Dam individual (0.191 and 0.199). Hence, despite its low incidence, interbreeding of the translocated fish with local Cotter River individuals contributed to the increased genetic diversity of the 2019 cohort (Table 2).

For Cotter‐only individuals born from 2016 onwards, average individual heterozygosity was lowest in years with low flow and highest in years of highest flow (Table 2, Appendix S6, Figure F2A), but the correlation between flow and mean heterozygosity was not significant (*p* > 0.05). Individual heterozygosity decreased across years of birth from 2016 to 2020 (LM1a *p* = 0.001, Adjusted *R* ^2^ = 0.02), but more variance was explained when cohorts were fitted as categories (LM1b *p* < 0.001; Adjusted *R* ^2^ = 0.068), with individuals born in 2018 having the lowest heterozygosity (significantly so compared to 2016 and 2017 cohorts; post hoc Tukey's test *p* < 0.05; Appendix S6, Table F2). Individual heterozygosity also decreased with distance to Cotter Reservoir (LM2 *p* < 0.001, adjusted *R* ^2^ = 0.051; Figure F2B), suggesting that the pool of breeders decreased in the upstream direction. Fish age at sampling was not significantly related to heterozygosity, providing no support for our expectation of higher survival of more heterozygous fish (LM3 *p* > 0.05; Figure F2C). Older individuals tended to be captured further from Cotter Reservoir (LM4 *p* < 0.001, adjusted *R* ^2^ = 0.084; Figure 2d).

### 3.6 Inbreeding depression for juvenile growth

**FIGURE 4:**
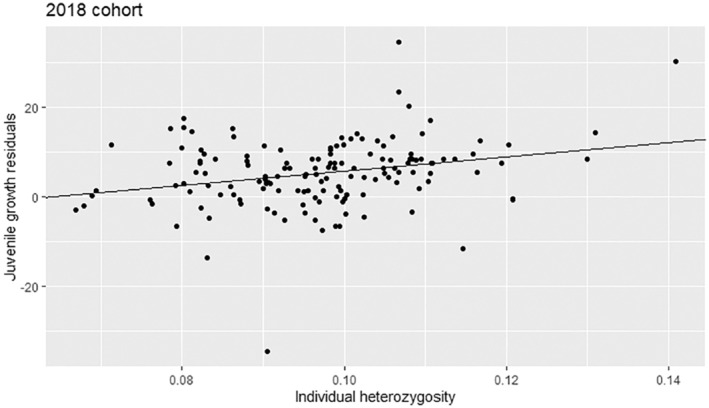
The plot of the linear model of growth residuals as a function of individual heterozygosity (PHt) for the 2018 cohort of Macquarie perch (LM6‐2018; *p* = 0.001; *R* ^2^ = 0.063).

Cohorts explained 24.6% of variance in growth residuals (LM5 *p* < 0.001). Individuals born in 2018 (predicted to be growing under the most stressful conditions) had the largest growth rates on average, followed by those born in 2019, with the smallest growth rate in individuals born in 2016, 2017 and 2020 (post hoc Tukey's tests *p* < 0.05; Table 2; Appendix S6, Table F3, Appendix S2, Figure B2). Individual heterozygosity alone did not predict growth residuals (LM6 *p* > 0.05), but the addition of cohort as a random effect significantly improved the model fit (ANOVA *t*‐test *p* < 0.0001). Growth residuals significantly increased with heterozygosity when the year of birth was controlled for (LMM6 *p* = 0.043), indicating that more‐inbred (less heterozygous) individuals grew slower than less‐inbred (more heterozygous) ones, in some years more than in others. Cohort 2018 appears to drive this relationship: inbreeding depression for juvenile growth was suggested by a significantly positive relationship between heterozygosity and growth residuals for this cohort (LM6‐2018 *p* = 0.001, adjusted *R* ^2^ = 0.063; Figure 4). No other birth cohorts showed significant effects of inbreeding depression (results for all models in Appendix S6, Table F3).

### 3.7 The number of effective migrants needed to reduce inbreeding to *F* = 0.1

Based on heterozygosity averaged across Cotter time/birth year cohorts (mean PHt_Cotter_) of 0.089, and heterozygosity averaged across three groups of translocated Cataract Dam fish (mean PHt_Cataract Dam_) of 0.144 (Appendix S6, Table F1), population‐level inbreeding of the Cotter Macquarie perch was *F*_Cotter_ = 1 − (0.089/0.144) = 0.382: almost twice the threshold level of 0.2 at which strong inbreeding depression is observed in wild populations. Assuming a target inbreeding level of *F*_augmented_ = 0.1, the estimated proportion of the augmented population derived from unrelated migrants is *f*_Cataract Dam_ = 1−sqrt(0.1/0.382) = 0.488. Based on the average number of breeders per year *N*_b_ = 42 (LD*N*_e_ $N_{b_{\mathrm{adj}}}$ averaged across cohorts; Table 2), the number of successful Cataract immigrants needed to reduce inbreeding to 0.1 is then *N*_m_ = 0.488 × 42/(1–0.488) = 40. If the annual number of breeders is averaged from *Colony2* estimates (*N*_b_ = 26) then *N*_m_ = 25. In either case, the number of effective migrants should approximately match the number of local effective breeders.

## 4 DISCUSSION

Here, we applied an adaptive management framework in a case of genetic rescue of the small, isolated and inbred Cotter River population of Macquarie perch, an endangered Australian freshwater fish, using a closely related lake population as a source. Inbreeding depression predicted for the Cotter population in Pavlova, Beheregaray, et al. (2017) is inferred here for a fitness trait (juvenile growth) expressed in stressful low‐flow conditions, highlighting the importance of continuing translocations to alleviate the loss of adaptive potential, inbreeding and inbreeding depression in this population. We further documented low reproductive outcomes of genetic augmentation through the translocation of 71 individuals from a related and genetically more diverse population, where only 0.45% (two of 446) of locally born Cotter individuals were genetically admixed, both offspring of the same translocated parent. Whereas our findings show that fish from the two sources can successfully interbreed under local conditions and increase genetic diversity, they also highlight that extending translocations and diversifying sources to include river populations need to be considered, to address environmental maladaptation as a possible reason for low success. This result mirrors findings from another system that fish stored for only a few generations in a lake might adapt to lake conditions such that they do not make the best colonists for rivers (Lutz et al., 2021). In addition, we detected positive effects of river flow on the number of breeding pairs, offspring genetic diversity and juvenile dispersal, which flags the importance of incorporating river flow regulation as an integral part of conservation of this species (see *Management recommendations* section below). Our findings of decline of genetic diversity and increase in age of sampled individuals with distance from the Cotter Reservoir, and stronger dispersal in downstream than upstream direction, suggest that Macquarie perch has poor upstream juvenile dispersal capacity in the Cotter River and that the riverine population upstream of Vanitys, which was extinct by the 1980s (Lintermans, unpublished data), might be still recovering via the upstream movement of adults through the recently‐installed fishway at Vanitys. It appears that barriers are still limiting breeder recolonization, especially in low‐flow years. This suggests that environmental flow regulations need to be reassessed considering Macquarie perch requirements for upstream spawning movement. In addition, translocating individuals of Cotter origin and from other sources to the most upstream sites may increase recruitment and facilitate genetic rescue. We estimated the target number of effective migrants needed to reduce population inbreeding to the level when short‐term population survival is no longer threatened by extinction through inbreeding depression and show that genetic augmentation needs to continue into the near future until at least 39 additional migrants successfully breed in the Cotter River. Genetic monitoring will be required to assess which management actions and gene flow sources lead to faster recovery of the Cotter River Macquarie perch population.

Obtaining fitness data for wildlife is often logistically challenging and may require decades for long‐lived species (Harrisson et al., 2019; Zilko et al., 2020). This long timeline is unacceptable because management inaction can rapidly drive populations to extinction through inbreeding depression and low capacity to recover from stochastic extreme events, such as droughts or fire. On the contrary, genetic data provides a reasonable proxy for genetic health and can be obtained relatively rapidly. Our study presented an opportunity to test for inbreeding depression for body size, found in other fish (Kincaid, 1983; Thrower & Hard, 2009). Smaller juveniles are expected to be less competitive and on a trajectory to have lower reproductive success and thus lower overall fitness (Vega‐Trejo et al., 2016). Smaller juveniles will also have reduced swimming speeds and remain longer in the predation range of gape‐limited predators such as trout (Ebner et al., 2007; Starrs et al., 2011). Our analyses of growth residuals for the Cotter River Macquarie perch indicate that this riverine population may experience inbreeding depression expressed in slower juvenile growth of inbred individuals during stressful conditions. Inbreeding depression was inferred only for the fish of 2018 cohort, which experienced low flow in their birth year and for a year after hatching, which could have increased other stressors (e.g. water temperatures and oxygenation, sedimentation). This cohort had the lowest genetic diversity (and thus the highest level of inbreeding) among the five studied cohorts, which may have made inbreeding depression easier to detect. Stressful conditions have been reported to exacerbate the effect of inbreeding on fitness in some species (Gallardo & Neira, 2005; Zajitschek & Brooks, 2010) but not others (Schons et al., 2022; Vega‐Trejo et al., 2016). For example, lower survival of inbred juvenile Coho salmon (*Oncorhynchus kisutch*) was expressed only in high‐density competitive environments (Gallardo & Neira, 2005).

River flow impacts many aspects of fish biology, including population and individual growth and recruitment (Humphries et al., 2020; Tonkin, Kearns, et al., 2017; Tonkin, Kitchingman, et al., 2017). In Macquarie perch, low flow can reduce egg and YOY survival, stem population growth and restrict or preclude passage of adults to spawning habitats (Broadhurst et al., 2019; Lintermans, 2023b; Tonkin et al., 2019). Extreme low flow that precludes access to spawning sites has been postulated as the cause of local extinction of Macquarie perch in the nearby Queanbeyan River (Lintermans, 2013a). Environmental flow releases in the Cotter River are aimed to maintain ecological values and functions, with maintenance of the Macquarie perch population a key value. Some of the considerations relevant to Macquarie Perch include maintaining natural flow profile (thermal and volume) where possible, release of riffle maintenance flow (150 ML/day for three days) during spring to prepare breeding sites and provide movement opportunity, provision/planning for pool‐flushing flows to reduce sediment accumulation and maintain pool refuge habitats, and reducing the rate of change in flow from 50% per day to 25% per day to minimize stranding of eggs. Furthermore, growth rates in fish often change in response to variation in population density, decreasing at high densities and increasing at low ones (Lorenzen & Enberg, 2002). The years of lowest flow in the Cotter River (2018 and 2019) were associated with the lowest number of Macquarie perch breeders, presumably leading to low population densities, and occurred during a severe drought in NSW and the Murray‐Darling Basin which had the driest 36 month period on record (BoM, 2020; Lintermans, 2023a). However, the cohorts born in these lowest‐flow years had significantly higher growth rates compared to the other cohorts (Figure F2). In the Dartmouth Macquarie perch population, growth rates increased with the reduction of competition due to rapid increases in resource availability during the refilling of the reservoir (Tonkin et al., 2014). Thus it might be possible that reduced recruitment in the Cotter River resulted in increased resource availability due to reduced intraspecific competition. Alternatively, it might be possible that stressful conditions impose stronger selection for faster growth and maturation. Regardless, frequent low‐flow conditions are likely to lead to fewer breeders and increased inbreeding in the Cotter River, and should be avoided. In contrast, high flow appeared to improve recruitment and genetic diversity and facilitate juvenile dispersal.

One might argue that our assumption of stronger growth being a positive fitness indicator might be incorrect, because in stressful environments smaller size may confer fitness benefits, for example, because smaller individuals require less resources to survive (Dupont‐Prinet et al., 2010). In such temporarily changed adaptive landscapes, being small might be more important than being outbred. However, in the predator‐saturated rivers such as the Cotter, where introduced trout (the likely major predator of juvenile Macquarie perch) are abundant, smaller slow‐growing individuals are at higher predation risk from gape‐limited predators for extended time periods, and so slow growth is unlikely a fitness benefit. Temporal selection for inbreeding as a survival strategy in stressful conditions is expected to be harmful to long‐term fitness under extremely variable conditions such as in the rivers of the Murray‐Darling Basin, where selection for small size would change very rapidly once flow increased. Our observation that the mean growth residuals are significantly higher for the 2018 cohort compared to the others (Table 2; Figures B2 and F2A) is not expected under a model of strong selection for small size in poor conditions, although environmental and genetic factors may interact to determine growth rates in complex ways. Overall, we consider that inbreeding depression for juvenile growth in stressful conditions may contribute to the observed relationship between inbreeding and growth during low‐flow conditions in our system (Cheptou & Donohue, 2011), but we acknowledge that environmental conditions (e.g. resource heterogeneity) can affect individual growth rate more strongly than does inbreeding (Schons et al., 2022).

The decline of genetic diversity with distance from the Cotter Reservoir detected in our study highlights the importance of physical connectivity between breeding sites for maintaining genetic connectivity. Acoustic telemetry data for Macquarie perch from Cotter Reservoir and another lacustrine population, Lake Dartmouth, show that resident lake fish migrate to spawning reaches of connected rivers annually and return to the lakes after breeding, where they can move large distances (>500 m/day) throughout the year (Thiem et al., 2013; Tonkin et al., 2018). The very small contribution of sampling sites to genetic structure, low genetic difference between river and reservoir sites and capacity to cover large geographic distances by dispersing fish in high‐flow years argues against distinct ‘river’ and ‘lake’ subpopulations in the Cotter. The almost twofold higher gene flow in the downstream than upstream direction and the tendency for the upstream sites to harbour older fish (with juveniles presumably moving downstream) support the Cotter Reservoir as a mixing ground and the role of barriers in restricting upstream movement and reducing upstream genetic diversity.

It is possible that the lentic conditions of the Cotter Reservoir create relaxed selective pressures compared to the Cotter River, as was also hypothesized for the fish sourced from Lake Dartmouth, who had surprisingly low survival and recruitment when translocated to the Ovens River (Lutz et al., 2021). Although our full‐sibling family data did not support the Cotter Reservoir as a source of migrants, sampling from the reservoir was limited. Meanwhile, larger numbers of breeders in high‐flow years may reflect improved access to spawning sites for downstream‐residing fish (Broadhurst et al., 2019), rather than improved breeding conditions for local fish. It may simply be that full colonization of the Cotter River up to Bendora Dam since the 2001 installation of Vanitys fishway has not yet occurred, or that in‐stream barriers have limited colonization.

Consistent with previous estimates, the number of breeders per reproductive cycle in the Cotter River, and effective population size per generation within the Cotter River was far lower than the threshold of 100 required for avoiding harmful inbreeding and 1000 required to maintain local adaptation (Pavlova, Beheregaray, et al., 2017), even after adjustment of the LD*N*_e_ estimates for age structure (Table 2). Together with the evidence of inbreeding depression, these results highlight the need to continue translocations and genetic augmentation in this Macquarie perch population and call for genetic management of small populations in other species. Although an annual number of breeding adults *N*_b_ estimated by *Colony2* were consistently lower than those by LD*N*_e_, comprising 43%–79% of estimated (${\hat{N}}_{b}$) and 28%–72% of adjusted LD*N*_e_ values, confidence intervals with ${\hat{N}}_{b}$ largely overlapped (Figure 3a). Because we used *P*_crit_ = 0.01 (and not 0.05) for LD*N*_e_ estimates, our adjusted *N*_b_ are likely to be upwardly biased (Waples et al., 2014).

Although LD*N*_e_ *N*_b_ estimates better tracked river flow, *Colony2* estimates appeared to be more robust to violations of the no‐migration assumption. For the two common sites dataset, LD*N*_e_ provided a lower estimate of the number of breeders for the 2019 cohort when the two admixed individuals were included (Figure 3b). This was the opposite of our expectation because at least one additional breeder contributed to the admixed fish. This pattern was not observed on the larger all‐samples dataset (Figure 3a) because rare Cataract alleles present only in one admixed individual (of 119) would have been ignored under the *P*_crit_ = 0.01 setting, resulting in the same estimate with or without the admixed individuals for the 2019 cohort. In contrast, the sibship assignment method in *Colony2* yielded larger or equal values of *N*_b_ when admixed individuals were included. This indicates that LD*N*_e_ is sensitive to violating the assumption of closed populations, and may not be appropriate for tracking dynamics of effective population size during genetic rescue, whereas *Colony2* might still yield reasonable values. Although LD*N*_e_ was found to perform well in simulated migration scenarios with low migration rates, methods of *N*_e_ estimation that explicitly account for migration, such as the moment method of *MLNe v1.0* (Wang & Whitlock, 2003) could be preferable (Gilbert & Whitlock, 2015).

Genetic augmentation is expected to alleviate inbreeding depression and elevate populations' capacity to adapt to changing environments through reduced frequencies of deleterious homozygotes and introduction of new potentially adaptive genetic variation (Ralls et al., 2020). One concern often raised about its implementation is the risk of genetic swamping—the replacement of the distinctive variation of the target population with variation in the source, due to the higher fitness of the introduced migrants (Zilko et al., 2021). Cautiousness about genetic swamping, along with the short supply of adults needed to achieve genetic rescue in the short timeframe of the project and limited capacity to provide quarantine, were considerations behind the relatively small number of Cataract individuals released in the current study. Given the historical gene flow between the Cotter and Murrumbidgee populations of Macquarie perch (Pavlova, Beheregaray, et al., 2017), genetic augmentation of the Cotter from Cataract Dam (historically sourced from the Murrumbidgee) is very conservative and poses negligible risk of swamping. Even if other, potentially differently adapted sources from the Murray‐Darling Basin, are used in the future, they will most likely mix successfully with the local fish, given the apparently unproblematic admixture observed elsewhere of much more distantly related inland and coastal Macquarie perch lineages (Lutz et al., 2022).

Meanwhile, evidence is accumulating to suggest that isolated reservoir populations (e.g. Cataract Dam) might be poor choices for translocations due to potentially having accumulated deleterious mutations that are maladaptive in new environments (Lutz et al., 2021; Wilder et al., 2020). Previous translocation attempts of Macquarie perch from Cotter Reservoir to Bendora Dam and the upper Cotter and Molonglo rivers, and from Googong Reservoir to the upstream Queanbeyan River appear to have failed for various reasons but reduced fitness may have contributed (Lintermans, 2013a, 2013b). In comparison, translocations of Cataract Dam Macquarie perch in 2017–2019 showed potential for success by 2021. It is worth noting that previous studies relied on the detection of recruits (YOY) to assess the success of translocations, whereas in the current study genetic monitoring was successfully applied, with sampling conducted several years post‐translocation. Genetic monitoring provided highly resolving information on several aspects of species biology and allowed us to detect that only two (0.45%) fish born after translocations had admixed ancestry and only one (1.5%) translocated fish was identified to have bred in the Cotter River. Translocations occurred over 3 years in which river flow ranged from extremely low (2018–2019) to moderate (2017) when opportunities for breeding with local fish were low. Using a mean *N*_e_/*N* ratio of 0.17, estimated for Macquarie perch based on observed allele frequencies using simulations (Pavlova, Beheregaray, et al., 2017) we would expect ~12 Cataract fish to be effective breeders (5, 5 and 2 effective breeders for 31, 28 and 12 fish translocated in 2017, 2018 and 2019, respectively). Compared with our estimates of the number of Cotter breeders for these years at Spur + Vanitys (LD*N*_e_ $N_{b_{\mathrm{adj}}}$: 21, 20, 13; *Colony2*: 12, 10, 9, respectively; Table 2), Cataract Dam fish should have comprised a reasonable proportion of breeders (14%–20% of all Spur+Vanitys breeders according to LD*N*_e_ estimates, or 18%–32% according to *Colony2 N*_b_ estimates). Low mate availability (Allee effect) could have contributed to the low reproductive success of Cataract fish (Lintermans, 2013a). However, the sole Cataract fish that was detected breeding in the Cotter River was translocated in 2019 and bred in the same year when the lowest number of local breeders contributed to local recruitment. Alternatively, in the Cotter River, the species matures at a larger size (140–300 mm) compared to in Cataract Dam (Lintermans, 2023a; Stocks et al., 2020), and the smaller sizes of the translocated fish (mean length 155 mm) could have made them unattractive mates compared to larger local competitors. Although the process of mate selection in Macquarie perch has not been studied, in many other fish species, larger individuals are more likely to be successful mates, as they are preferred by females, more competitive during male–male aggression and able to produce more sperm (Kim et al., 2021). Experimental feeding of Cataract fish in captivity showed that fish rapidly grow when resources are abundant and predation pressure is released (Stocks et al., 2020). Thus, it is possible that recruitment of translocated Cataract fish will improve after fish have grown and acclimated to the novel environment. Successful recruitment of translocated Macquarie perch in the Queanbeyan River was detected only more than a decade after the translocations (Lintermans, 2013a).

It is possible that some translocated individuals died before having an opportunity to breed. Lower survival of translocated fish compared to local ones has been reported for some species (Monk et al., 2020), and low flow could have contributed to stress (Ruhí et al., 2016). Alternatively, the reservoir‐sourced translocated individuals could have moved to the Cotter Reservoir where habitats are more familiar, as seen in eel‐tailed catfish *Tandanus tandanus* (Carpenter‐Bundhoo et al., 2020). Finally, Cataract Dam fish could be maladapted to river conditions, causing reduced survival and breeding performance. Adaptation to local conditions or loss of adaptive traits under relaxed selection can occur very rapidly (Bell et al., 2004; Lahti et al., 2009). Although originally sourced from Murrumbidgee River, Cataract Dam fish dwelt in lake environments for 7–14 generations (Lutz et al., 2022). Maladaptation of lake‐residing fish to river environments was hypothesized to explain the low performance of Macquarie perch translocated from Lake Dartmouth to the Ovens River, compared to pure or admixed descendants of Yarra River fish, which had demonstrably higher survival, juvenile growth and reproduction after stocking (Lutz et al., 2021). By analogy, even if the translocated Cataract fish themselves are less fit in the Cotter than are local breeders, their admixed offspring with increased genetic diversity would be expected to do better.

## 5 MANAGEMENT RECOMMENDATIONS

Inbreeding depression detected in the Cotter Macquarie perch population highlights the urgent need for genetic rescue to increase population fitness, alleviate inbreeding and increase population adaptive potential. Although translocations from Cataract Dam resulted in some interbreeding and slightly increased genetic diversity in the Cotter, additional translocations are needed to reduce inbreeding to *F* = 0.1. Even if currently translocated individuals increase their breeding contribution from the current <3% (one Cataract breeder over 44 total breeders estimated by Colony2 for Spur + Vanitys) to the expected 17% (as per the estimated ratio of *N*_e_/*N* = 0.17), the total number of migrants needs to be 40/0.17 = 235. Thus, at least as many additional migrants will be required to reduce the observed inbreeding to the target value. Thus, we recommend to translocate ~150 additional individuals as soon as practical and reassess the need for additional translocations after genetic monitoring several years later. Translocating mature fish is likely to facilitate their survival and breeding after translocation, and moving fish >3 months prior to the start of the breeding season to reduce the effect of capture and handling (Rowland, 1988) may provide sufficient time for adjustment to local conditions while encouraging individuals to stay in the river for breeding. In low‐flow years, releasing mature individuals into the upstream sites could help alleviate challenges to fish accessing those sites and upstream inbreeding. Nevertheless, preference should be given to sites that have microhabitats similar to those of the source populations, to maximize the survival and reproduction of translocated individuals (Carpenter‐Bundhoo et al., 2020). If smaller‐sized fish are moved, then releasing them across multiple river sites could decrease local competition. Diversifying translocation sources for additional translocations, including river sources such as the Murrumbidgee, Yarra and Ovens Rivers, should be considered. Provided a reasonable number of fish could be sourced from each population, using multiple sources will allow for less pressure on a single source population and provide the target population with more genetic diversity to build resilience to environmental changes (Mitchell et al., 2022). Continuing genetic monitoring of the Cotter River will be essential to assess to what extent Cataract Dam fish (and future translocations) contribute to recruitment and admixture, and whether some sources are better suited to Cotter conditions than others. Intensifying genetic monitoring of the Cotter Reservoir fish will allow testing whether it acts as a sink for translocated, potentially lake‐adapted, fish, as well as Cotter River fish. We currently do not recommend moving fish directly into the Cotter Reservoir, as the contribution of the reservoir fish to the riverine Macquarie perch population it is not well understood. The current riverine population has developed from natural upstream dispersal following the installation of fishways at Vanitys and subsequently Pipeline. However, there are still numerous barriers present within the river (Broadhurst et al., 2016) which in conjunction with low flow may be limiting riverine *N*_e_ by restricting the extent, rapidity and number of breeders colonizing the river. Assisted dispersal of Cotter riverine subadults and adults to upstream sites may facilitate a more rapid increase in riverine *N*_b_ and *N*_e_, as well as help alleviate Allee effect.

Importantly, dependence on the number of breeding individuals, offspring genetic diversity and dispersal on water flows highlights the importance of providing adequate flows, in addition to genetic augmentation, for improving the health of this Macquarie perch population. For the Cotter Macquarie perch, we advise that the provision of targeted increased flows is required to facilitate upstream movement and reduce stressful low‐flow habitat conditions because the health of the population depends on the number of breeding individuals, offspring genetic diversity and dispersal, all of which were shown to be linked to flows in the river. For example, the provision of a larger flow in early October prior to the spawning season to drown out barriers during the spawning run should be considered. This could be achieved through the reallocation of one of the bimonthly 150 ML/day releases to maintain this level of flow for October and November. In years of low flow, such as 2018, additional releases should be considered. We stress that even if adjusting flow regulation alone facilitated population growth initially, without genetic augmentation, the Cotter population will still be at risk of extinction through inability to adapt, loss of genetic diversity and inbreeding depression (Pavlova, Beheregaray, et al., 2017).

## 6 CONCLUSIONS

Genetic augmentation and genetic rescue are increasingly important conservation management tools for improving the population health of small, fragmented populations of threatened species. Given that climate change is expected to result in warmer and harsher environments, mitigating inbreeding and inbreeding depression will be essential for preventing the extinction of small populations. Genetic monitoring is an efficient way of assessing the contribution of translocated individuals to recruitment and admixture in target populations, and should routinely be performed to assess population health and assist in planning management actions. Having a clear target for the reduction of inbreeding helps to plan the number of translocated individuals required, whereas understanding drivers of recruitment and dispersal (such as flow) helps to plan timing and release locations. This study adds a test case of evidence‐based planned and monitored genetic management. It substantiates the need to use larger numbers of mature individuals during translocations, using multiple sources including rivers, targeting upstream sites that are less accessible by fish during lower flows and modifying river flow regulation to improve upstream fish passage during breeding seasons, followed by genetic monitoring after several seasons of breeding opportunities to understand the outcomes of different strategies.

## CONFLICT OF INTEREST STATEMENT

We declare no conflict of interest.

## Supporting information


Appendix S1

Data S1

## Data Availability

All data used in this study, R scripts for analyses and Supplementary Material S1 are available on Bridges data repository at https://doi.org/10.26180/19376570. Benefit sharing statement: Benefits from this research accrue from the sharing of our data and results on public databases as described above.
