# Supplementary material for: Planning and implementing genetic rescue of an endangered freshwater fish population in a regulated river, where low flow reduces breeding opportunities and may trigger inbreeding depression
Source: Evol Appl. 2024 Apr 11;17(4):e13679. doi: 10.1111/eva.13679 (PMC11009430; doi:10.1111/eva.13679)
Supplement: Supplementary file 1 — Appendix S1 [file EVA-17-e13679-s002.docx]

**Appendices for the manuscript:**

Pavlova, A., Schneller, N., Lintermans, M., Beitzel, M., Robledo-Ruiz, D., Sunnucks, P., 2024. **Planning and implementing genetic rescue of an endangered freshwater fish population in a regulated river, where low flow reduces breeding opportunities and may trigger inbreeding depression.**

All ***R scripts*** and ***data files*** used for the analyses are available in Bridges data repository at DOI <https://doi.org/10.26180/19376570>. File ***Cotter_Cataract.genetics.finess.csv*** contains field data and genetic results.

**Appendix S1.** Details on Cotter River barriers, field sampling and fish length distributions. Geographic distances and number of barriers among Cotter River field sites (Table A1), sample sizes for genetic samples collected across years (Table A2) and distribution of total lengths (Fig. A1) and inferred ages at sampling per cohort (Fog. A2) for Macquarie perch collected in the Cotter River.

Vanitys Crossing—a ford constructed in 1970s—precluded access of Macquarie perch to upstream spawning habitat for three decades, effectively restricting the population to the ~5.5 km downstream section of the river and Cotter Reservoir (Ebner and Lintermans 2007; Ebner et al. 2008). Construction of two fishways facilitated passage of Macquarie perch to suitable upstream habitat, at Vanitys since 2001 and Pipeline since 2012 (Ebner et al. 2008). The Cotter Reservoir was enlarged in 2013, inundating previous downstream spawning sites used by reservoir fish (here included in the Cotter Reservoir site), which prohibited breeding for the next three years, because barriers prevented fish migration to the next upstream spawning sites (Broadhurst et al. 2016). Cotter Reservoir fish resumed breeding after the enlarged reservoir filled in 2016, inundating the in-stream barriers and providing more consistent access to the Condor spawning site.

**Table A1.** Geographic distances (km, below diagonal) and number of barriers between sampling sites (above diagonal; Broadhurst et al. (2016)). *Geographic distances* between neighbouring sites (upstream to downstream): 7.Burkes Ck Crossing 🡪 3.9 km 🡪 6.Pipeline Rd Crossing 🡪 4.85 km 🡪 5.Spur Hole 🡪 2.85 km 🡪 4.Vanitys Crossing 🡪 1.6 km 🡪 3.Motherhole 🡪 0.4 km 🡪 2.Condor Ck 🡪 0.3 km 🡪 1.Cotter reservoir (upstream end of impoundment). *Barriers to movement* (M - medium, L - large) between neighbouring sites are: 7.Burkes Ck Crossing 🡪 3M+2L 🡪 6.Pipeline Rd Crossing 🡪 8M+3L 🡪 5.Spur Hole 🡪 2M 🡪 4.Vanitys Crossing 🡪 2M 🡪 3.Mother Hole 🡪 1M 🡪 2.Condor Ck 🡪 11M+7L 🡪 1.Cotter Reservoir. The number of barriers between Cotter Reservoir and Condor Ck depends on the water level of the Cotter Reservoir, being largest for the lowest levels seen in 2018.

|  | **1.Reservoir** | **2.Condor** | **3.Motherhole** | **4.Vanitys** | **5.Spur** | **6.Pipeline** | **7.Burkes** |
| --- | --- | --- | --- | --- | --- | --- | --- |
| **1.Reservoir** | - | 18 | 19 | 21 | 23 | 34 | 39 |
| **2.Condor** | 0.30 | - | 1 | 3 | 5 | 16 | 21 |
| **3.Motherhole** | 0.70 | 0.40 | - | 2 | 4 | 15 | 20 |
| **4.Vanitys** | 2.30 | 2.00 | 1.6 | - | 2 | 13 | 18 |
| **5.Spur** | 5.15 | 4.85 | 4.45 | 2.85 | - | 11 | 16 |
| **6.Pipeline** | 10.00 | 9.70 | 9.3 | 7.70 | 4.85 | - | 5 |
| **7.Burkes** | 13.90 | 13.60 | 13.2 | 11.60 | 8.75 | 3.90 | - |

**Table A2.** Sampling details for Macquarie perch genetic rescue in the Cotter River: timing of translocations from Cataract Dam to the Cotter River, and number of genetic samples collected during monitoring in the Cotter River (including samples that failed to produce genotypes of sufficient quality).

| **Sampling years** | **Translocations from Cataract Dam** | **Cotter monitoring** | | **Total** |
| --- | --- | --- | --- | --- |
|  |  | **Non-lethal fin-clip sample** | **Lethal larval sample** |  |
| 2002 |  | 3 |  | 3 |
| 2006 |  | 1 |  | 1 |
| 2007 |  | 13 |  | 13 |
| 2011 |  | 51 |  | 51 |
| 2013 |  | 18 |  | 18 |
| 2016 |  |  | 20 | 20 |
| 2017 | 31 |  | 24 | 55 |
| 2018 | 28 | 125 | 22 | 175 |
| 2019 | 12 | 105 | 48 | 165 |
| 2020 |  | 98 | 21 | 119 |
| 2021 |  | 67 |  | 67 |
| **Total** | **71** | **481** | **135** | **687** |

**Table A3.** Number of samples that yielded good quality genotypes collected each year from 2002 to 2021 across seven sampling sites.

| **Sampling sites** | **Sampling years** | | | | | | | | | | |  |
| --- | --- | --- | --- | --- | --- | --- | --- | --- | --- | --- | --- | --- |
|  | **2002** | **2006** | **2007** | **2011** | **2013** | **2016** | **2017** | **2018** | **2019** | **2020** | **2021** | **Total** |
| 1.Reservoir | 3 | 1 | 13 | 5 | 8 |  |  |  | 13 |  |  | 43 |
| 2.Condor |  |  |  |  |  | 10 | 16 | 18 | 1 |  |  | 45 |
| 3.Motherhole |  |  |  |  |  |  |  | 33 | 16 | 14 | 2 | 65 |
| 4.Vanitys |  |  |  | 24 | 8 | 8 | 4 | 41 | 23 | 28 | 31 | 167 |
| 5.Spur |  |  |  | 20 | 2 | 2 |  | 36 | 76 | 66 | 21 | 223 |
| 6.Pipeline |  |  |  | 1 |  |  |  | 13 | 14 | 11 | 13 | 52 |
| 7.Burkes |  |  |  |  |  |  |  | 6 | 9 |  |  | 15 |
| Cataract Dam |  |  |  |  |  |  | 28 | 28 | 12 |  |  | 68 |
| **Total** | **3** | **1** | **13** | **50** | **18** | **20** | **48** | **175** | **164** | **119** | **67** | **678** |

**Figure A1**. Distribution of total lengths of 455 individual Macquarie perch captured during monitoring of the Cotter River and Reservoir from 2016 onwards. The first peak corresponds to measured larvae collected during December snorkelling surveys (length <20 mm). Other peaks correspond to individuals sampled in February-March surveys (with the exception of 8 young-of year fish length 52-73 mm, collected in May, June and October). The second peak represents older young-of year (YOY, 35-75 mm), the third - individuals older than 1YO but younger than 2YO (1-2YO, 85-150 mm). Of the 12 remaining individuals (all sampled in 2021), those with length 160-185 mm were assigned to 2-3YO, and those with length 210-245 mm to 3-4YO. These age groups and capture dates were used to assign individuals to five birth-year cohorts, 2016 to 2020. Appropriateness of the assignments (including 2-3YO and 3-4YO ones) was corroborated by sibship analysis detecting full-sib families with members belonging to the same birth cohort. Fish sampled before 2016 were analysed together in their own category (referred as ‘time cohort’ adults 2002-2013), as length measurements were missing for many individuals. The range of recoded lengths (156-332 mm) indicated that this cohort included mature individuals.

**Figure A2.** Distribution of age categories sampled across five cohorts (numbers within each category are sample sizes)

**Appendix S2.** Estimating growth residuals for the inbreeding depression model (R script ***1.Analyses_of_field_data_age_length_Gompertz_growth_residuals.R*** and data in ***Cotter2016onwards_length.csv***).

A standard Gompertz non-linear regression model was fitted to length Lx (in mm) and age X (in years) of the Cotter fish with parameters adapted from Tonkin et al. (2017): $L_{x}=L_{\infty} e^{ e^{\left(-b\left(X-M\right)\right)}}$, where L*_x_* is the length at age *X*, L_∞_ is the asymptotic length, *b* is the instantaneous growth rate, and *M* is the inflection point of the curve. Age of each individual (in years) was calculated as follows: (date of sampling- inferred date of birth)/365. Date of birth was assumed to be November 30 of the inferred year of birth (based on inferred breeding season).

When the data was limited to Cotter fish with length < 150mm (<2YO), estimated growth parameters for were *Length ~ 113.96 * exp(-2.52 * exp(-4.19 * inf.age.at.capture))*. Growth residuals had a normal distribution, with variance increasing with age (Fig. B1). Growth residuals were larger for 2018-born fish compared to the remaining cohorts. Because log-transformed residuals appeared skewed, we used untransformed residuals for modelling inbreeding depression.

**Figure B1**. Distribution of growth residuals across inferred ages of fish, for larvae YOY from snorkelling surveys, older YOY and 1+ individuals. X-axis shows age (in years), y-axis shows growth residuals.


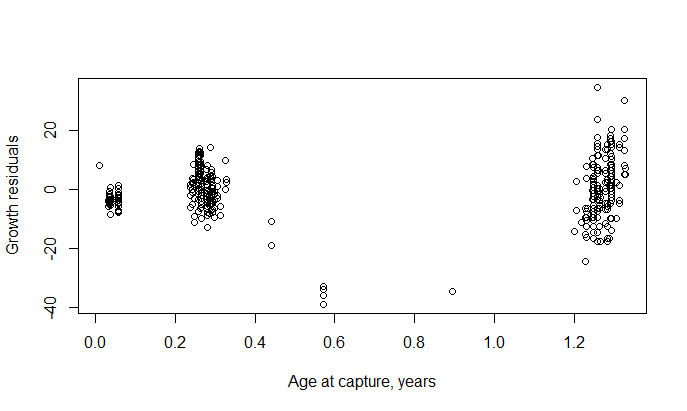


**Figure B2**. Growth residuals for five cohorts (post-hoc Tukey tests in **Table F3a**). Boxes (25th and 75th percentiles of distribution) are coloured according to non-significant results of the post-hoc Tukeys test: the same colour indicate cohorts that are not significantly different from each other (P<0.05). Line in the box is a median; the whiskers extend to the minimum and maximum values that are not considered outliers, individual points beyond the whiskers are outliers. Growth residuals of two admixed fish (born in 2019) were 8.5 for MP_L172 and -9.7 for MP_L204.


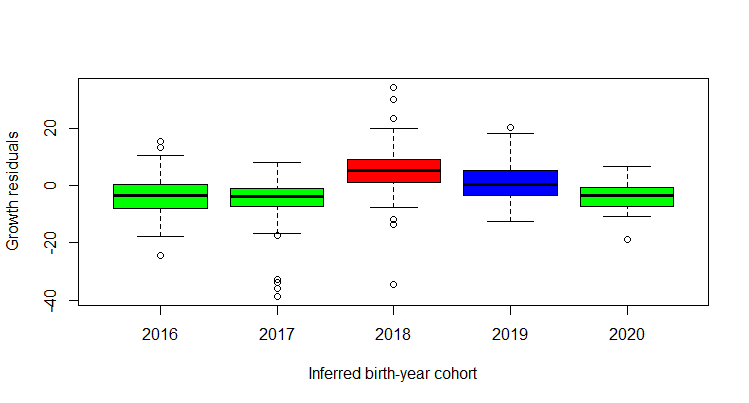


**Appendix S3.** Flow conditions (Fig. C1; R script ***4.Analyses_of_flow_Ne_disp.dist.R***, data in ***ACT_flow_data_Vanitys.csv***.) and relative abundance of the YOY in the Cotter River were used to predict the unfavourable years for juvenile Macquarie perch by the experts (MB and ML, respectively). Environmental flows from Bendora Dam are provided according to Icons licence guided by the ACT flow Guidelines. They are broken into four functional components: (1) Base flow –a variable monthly flow, (2) Riffle flow –150 ML/day every two months, (3) Pool flush –500 ML/day once every 2 years, and (4) Special purpose (irregular). Overflow from Bendora Dam (such as 2016 flows and those at the end of 2020) can dwarf these.

The 2018 season was predicted to be the worst based on lowest flow in the reach. Although 2019 was even drier in terms of rainfall - eflow release (and possibly transfer flows) resulted in flows of 200 ML/day which were not seen in 2018. Low flow were expected to reduce the potential for spawning migration through the system.

Based on surveys of YOY at five riverine sites in the Cotter (Broadhurst et al. 2020), 2016 had a poor recruitment of YOY at all sites. 2017 had average recruitment of YOY at Spur, Burkes and Vanitys and below average at Pipeline. 2018 had poor YOY recruitment at most sites. 2019 had better than average YOY recruitment at Spur and Burkes, and average at Vanitys and Pipeline. 2020 was a poor year for YOY everywhere except Pipeline.

**Figure C1.** Mean daily discharge (in ML per day) in the Cotter River from 2016 to 2020 (Icon Water, ALS hydrographic data, gauge 410725 at Vanitys Crossing Available at <http://www.bom.gov.au/waterdata/>). Red lines indicate per-year averages (Table 2 of the main manuscript).


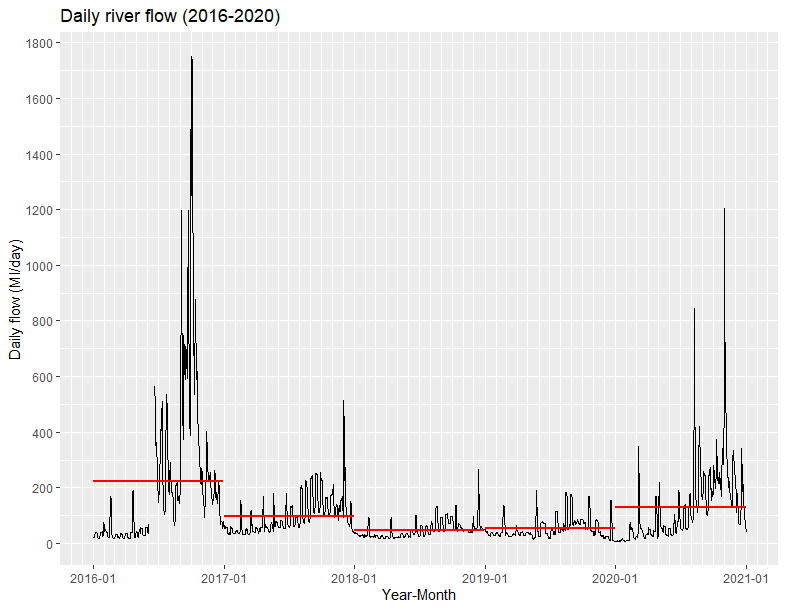


**Appendix S4.** Genetic structure using principle coordinate analyses (PCoA) (R script ***2.Analyses_of_DArT_genotypes.R***, data ***Report_DMacq21-6666_12_moreOrders_SNP_mapping_2.csv***. covariate file ***DMacq21-6666_covariate_CotterR_CatD.csv***). To conduct Principal Coordinate Analysis (PCoA) we used *Pcoa* function of *dartr* - a wrapper for *Pca* function of *adegenet*, which centres but does not scale genotypes prior to analyses; we also report percent variance explained when genotyped were centered and scaled, run through *glPca* of *adegenet* (scaling did not affect the structure). First PCoA axis of the on complete Cotter+Cataract dataset (Fig. 2A of the main manuscript) explained 18.4% of the total variance (12.3% when variables were scaled). PCoA of the Cotter-only dataset (Fig. 2B and C of the main manuscript) the first two axes explained 5.5% and 4.5% of all variance in the data (1.75% and 1.4% when variables were scaled). Separate analyses on each cohort (Fig. D1) showed limited genetic structure that varied across cohorts.

**Figure D1.** PC1 vs PC2 plots of separate PCoAs for the multi-age group (2002-2013) and five cohorts on re-filtered datasets (genotypes were centered, but not scaled). Note that each cohort comprises samples collected in different years, but inferred to have been born in the same year. Colours indicate sampling sites (rainbow from most downstream (red) to most upstream (purple); ellipses contain 95% of the fish from the same sampling site. *F*_ST_-values are results of AMOVAs.


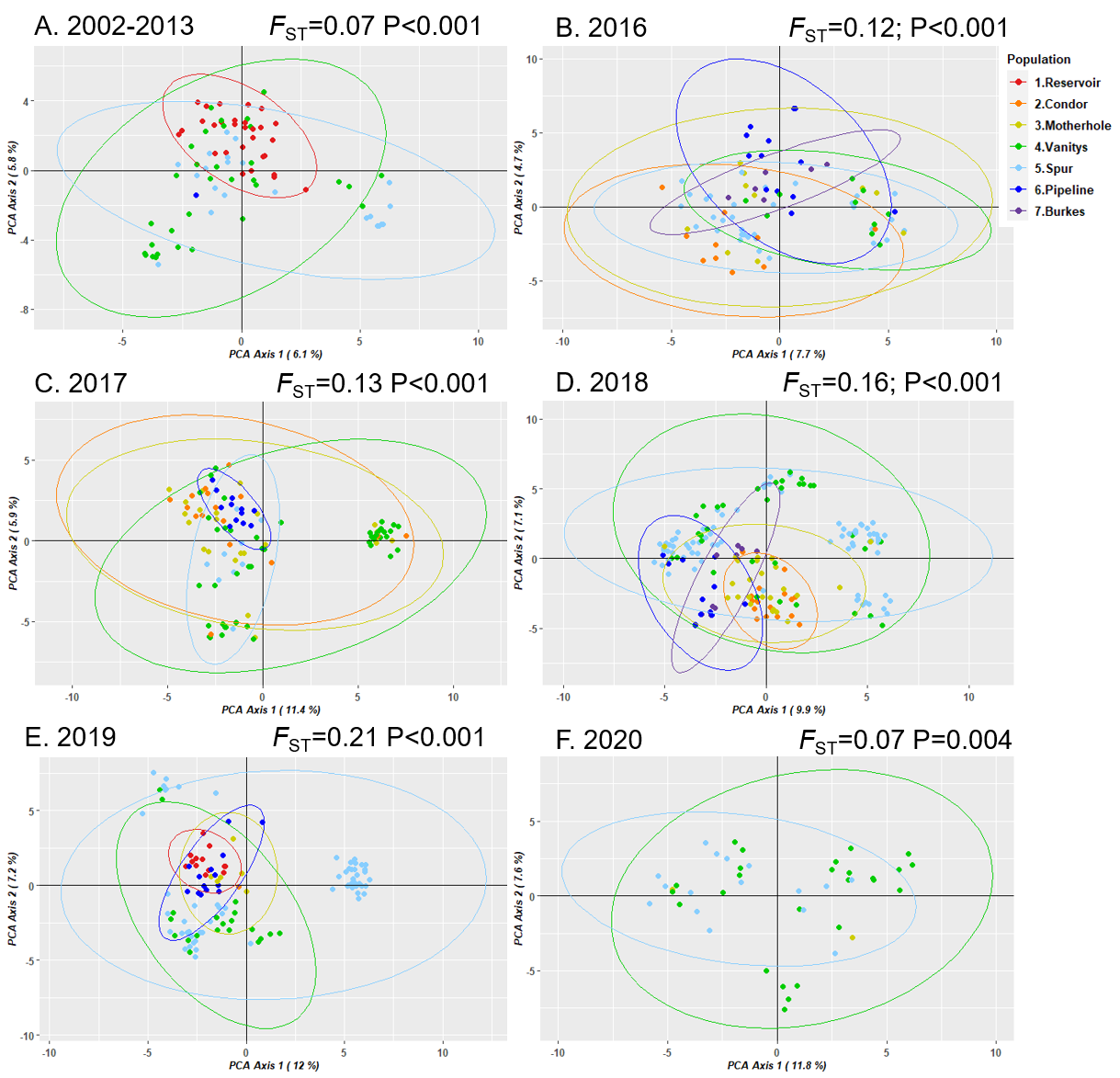


**Appendix S5.** Results of *Colony2* identity and full-sibship analysis of Cotter-only dataset, and analysis of juvenile dispersal.

***Identity analysis.*** Identity analysis detected ten pairs of identical genotypes, revealing eight individuals sampled two or three times (Table E1). One individual was inferred to have dispersed 6 km downstream from Burkes to Pipeline from 2019 to 2020. Three individuals were recaptured at the same location (Spur and Vanitys) one to two years apart; one of these, sampled two years apart, did not grow as much as expected leading to wrong year of birth inferred based on its length. In the other cases were either the same individual was sampled twice or identical twins were sampled at the same site within a short time span.

**Table E1**. Ten pairs of genotypes identified as clones, with inferences. Six genotypes that were included in full-sib families are highlighted in yellow.

| **Ind1** | **Ind1 site and birth cohort** | **Ind1 sampling date** | **Ind2** | **Ind2 site and birth cohort** | **Ind2 sampling date** | **Inference** |
| --- | --- | --- | --- | --- | --- | --- |
| MP_N189 | 7.Burkes2018 | 27/02/2019 | MP_L289 | 6.Pipeline2018 | 23/03/2020 | 1 |
| MP_N212 | 5.Spur2018 | 04/03/2019 | MP_L115 | 5.Spur2018 | 16/03/2020 | 2 |
| MP_N27 | 5.Spur2013 | 09/03/2011 | MP_N400 | 5.Spur2013 | 04/08/2011 | 2 |
| MP_N27 | 5.Spur2013 | 09/03/2011 | MP_N413 | 5.Spur2013 | 19/02/2013 | 2 |
| MP_N400 | 5.Spur2013 | 04/08/2011 | MP_N413 | 5.Spur2013 | 19/02/2013 | 2 |
| MP_N272 | 4.Vanitys2017 | 15/03/2019 | MP_L257 | 4.Vanitys2017 | 04/03/2021 | 2 |
| MP_N51 | 6.Pipeline2016 | 22/02/2018 | MP_N61 | 6.Pipeline2016 | 28/02/2018 | 3 |
| MP_N31 | 5.Spur2013 | 09/03/2011 | MP_N39 | 5.Spur2013 | 09/03/2011 | 3 |
| MP_L246 | 6.Pipeline2018 | 05/03/2021 | MP_L251 | 6.Pipeline2018 | 05/03/2021 | 3 |
| MP_L248 | 6.Pipeline2019 | 05/03/2021 | MP_L252 | 6.Pipeline2019 | 05/03/2021 | 3 |

^1^An individual sampled in 2019 dispersed 6 km downstream to the next site in one year.

- N189 (length 46 mm) aged as YOY in 2019 at Burkes, L289 (109 mm) aged as 1-2YO in 2020 at Pipeline.

^2^Three individuals were recaptured again once or twice at the same location, one to two years apart.

- N27 unknown age in 2011, N400 (183 mm) 2-3YO in 2011, and N413 (264 mm) >4YO in 2013 at Spur;
- N212 (53 mm) YOY in 2019, L115 (114mm) 1-2YO in 2020 at Spur;
- N272 (96 mm) 1-2YO in 2019, L257 (224 mm) 3-4YO in 2021 at Vanitys.

^3^The same individual was sampled twice or identical twins were sampled at the same site in short time span.

- N31 and N39 of unknown age on the same date in 2011 at Spur;
- L248 (113 mm) 1-2YO and L252 (113 mm) 1-2YO on the same date in 2021 at Pipeline;
- L246 (170mm) 2-3YO and L251 (169mm) 2-3YO on the same date in 2021 at Pipeline;
- N51 (116 mm) 1-2YO and N61 (121 mm) 1-2YO within 6 days in 2018 at Pipeline.

***Full-sibship analysis.*** Some but not all of these genotypes were also inferred to be full-siblings (Tables E2-E3). Family 14 (N=8) included MP_L257, but not MP_N272. Family 18 (N=7) included MP_L115, but not MP_N212. Family 48 (N=2) included MP_L248 and MP_L252. Family 73 (N=2) included MP_N27 and MP_N413, but not MP_N400. The remaining genotyped from Table E1 were not consistently assigned to a full-sib family by 3 or more replicate runs.

***Dispersal analysis.*** We analysed juvenile dispersal using data on maximum distance between full-siblings for 66 full-sib families of two or more individuals born from 2016 onwards (see script ***4.Analyses_of_flow_Ne_disp.dist.R*** and data in ***Families_dispersal_flow.csv***). When fitted alone in a linear model, water flow averaged per birth-year did not explain variation in maximum distance between siblings (P>0.05), both fitted together, flow and family size significantly explained 11.3% of the variance in maximum distance between full siblings (P<0.01): larger families (P<0.01) and higher flow levels (P<0.05) corresponded to larger maximum distance between siblings.

***Direction of dispersal.*** Of 25 families of five or more individuals born 2016-onwards (Appendix E Table E2), 24% did not disperse, 28% dispersed downstream, 16% dispersed in an upstream direction, and for 32% the direction was not possible to infer based on geographic distribution and sampling times of the siblings. For example, members of the most numerous family (family 1, 32 full-sibs born in 2019) were sampled at the same site (Spur) across 2019-2021, suggesting that juveniles may stay at their birthplace for the first two years of their lives. Members of the second largest family (family 2: 30 full-sibs born in 2018) were sampled at Spur (N=16) and Vanitys (2) in 2019, and at Spur (10), Vanitys (1) and Motherhole (1) in 2020, and therefore downstream direction was inferred. Similarly, members of the third largest family (family 3: 26 full-sibs born in 2017) were sampled at Vanitys (N=3) and Condor (2) in 2017, and at Vanitys (15) and Motherhole (5) in 2018. An example of upstream dispersal within one family is family 7: full-sibs born in 2018 were sampled in 2018 at Condor (N=6), in 2019 at Motherhole (3) and in 2020 at Vanitys (1).

**Table E2.** Distribution of full-siblings for 27 full-sib families with 5 or more members across sampling sites and sampling years, and the inference of direction of movement of some family members within the Cotter River. Here, ‘unclear’ direction includes cases (marked with asterisks) where some family members move downstream and some upstream, as well as those where direction of movement could not be determined. Samples collected in the birth year are larvae, those collected in the year after birth year are older young-of-year (YOY) etc. Families are numbered in descending order of number of family members (see ***Cotter_Cataract.genetics.finess.csv***). The most upstream site, Pipeline, is highlighted in yellow.

| **Family ID** | **Time/birth- year cohort** | **Inferred direction of movement** | **Site sampled** | **Year sampled** | | | | | | | **N inds per site per family** |
| --- | --- | --- | --- | --- | --- | --- | --- | --- | --- | --- | --- |
|  |  |  |  | 2011 | 2016 | 2017 | 2018 | 2019 | 2020 | 2021 |  |
| 1 | 2019 | None | 5.Spur |  |  |  |  | 22 | 6 | 4 | 32 |
| 2 | 2018 | downstream | 3.Motherhole |  |  |  |  |  | 1 |  | 1 |
|  |  |  | 4.Vanitys |  |  |  |  | 2 | 1 |  | 3 |
|  |  |  | 5.Spur |  |  |  |  | 16 | 10 |  | 26 |
| 3 | 2017 | downstream | 2.Condor |  |  | 2 |  |  |  |  | 2 |
|  |  |  | 3.Motherhole |  |  |  | 5 |  |  |  | 5 |
|  |  |  | 4.Vanitys |  |  | 3 | 15 |  |  |  | 18 |
| 4 | 2019 | downstream | 4.Vanitys |  |  |  |  |  |  | 5 | 5 |
|  |  |  | 5.Spur |  |  |  |  | 3 | 8 | 2 | 13 |
| 5 | 2018 | downstream | 3.Motherhole |  |  |  |  |  | 1 |  | 1 |
|  |  |  | 4.Vanitys |  |  |  |  | 2 |  |  | 2 |
|  |  |  | 5.Spur |  |  |  |  | 5 | 5 |  | 10 |
| 6 | 2018 | unclear | 4.Vanitys |  |  |  | 1 | 2 |  |  | 3 |
|  |  |  | 5.Spur |  |  |  |  | 7 | 3 |  | 10 |
| 7 | 2018 | upstream | 2.Condor |  |  |  | 6 |  |  |  | 6 |
|  |  |  | 3.Motherhole |  |  |  |  | 3 |  |  | 3 |
|  |  |  | 4.Vanitys |  |  |  |  |  | 1 |  | 1 |
| 8 | 2018 | None | 4.Vanitys |  |  |  | 2 | 5 | 3 |  | 10 |
| 9 | 2019 | downstream | 4.Vanitys |  |  |  |  |  | 1 | 1 | 2 |
|  |  |  | 5.Spur |  |  |  |  |  | 5 | 2 | 7 |
| 10 | 2016 | unclear* | 2.Condor |  | 1 |  |  |  |  |  | 1 |
|  |  |  | 3.Motherhole |  |  |  | 1 |  |  |  | 1 |
|  |  |  | 4.Vanitys |  |  |  | 1 |  |  |  | 1 |
|  |  |  | 5.Spur |  | 1 |  | 4 |  |  |  | 5 |
|  |  |  | 6.Pipeline |  |  |  | 1 |  |  |  | 1 |
| 11 | 2018 | unclear | 4.Vanitys |  |  |  | 1 |  | 3 |  | 4 |
|  |  |  | 5.Spur |  |  |  |  | 3 | 2 |  | 5 |
| 12 | 2020 | unclear | 4.Vanitys |  |  |  |  |  | 2 | 2 | 4 |
|  |  |  | 5.Spur |  |  |  |  |  | 3 | 1 | 4 |
| 13 | 2018 | None | 5.Spur |  |  |  |  | 1 | 7 |  | 8 |
| 14 | 2017 | unclear | 2.Condor |  |  | 1 |  |  |  |  | 1 |
|  |  |  | 4.Vanitys |  |  |  | 6 |  |  | 1 | 7 |
| 15 | 2020 | unclear | 3.Motherhole |  |  |  |  |  |  | 1 | 1 |
|  |  |  | 4.Vanitys |  |  |  |  |  | 1 | 1 | 2 |
|  |  |  | 5.Spur |  |  |  |  |  |  | 4 | 4 |
| 16 | 2019 | unclear | 4.Vanitys |  |  |  |  | 2 |  | 3 | 5 |
|  |  |  | 5.Spur |  |  |  |  |  | 2 |  | 2 |
| 17 | 2018 | downstream | 4.Vanitys |  |  |  |  |  | 1 |  | 1 |
|  |  |  | 5.Spur |  |  |  |  | 4 | 2 |  | 6 |
| 18 | 2018 | downstream | 4.Vanitys |  |  |  |  |  | 1 |  | 1 |
|  |  |  | 5.Spur |  |  |  |  | 3 | 3 |  | 6 |
| 19 | 2019 | None | 6.Pipeline |  |  |  |  |  | 4 | 2 | 6 |
| 20 | 2020 | None | 4.Vanitys |  |  |  |  |  | 3 | 3 | 6 |
| 21 | 2019 | upstream | 4.Vanitys |  |  |  |  | 3 |  | 2 | 5 |
|  |  |  | 5.Spur |  |  |  |  |  |  | 1 | 1 |
| 22 | 2018 | unclear | 4.Vanitys |  |  |  |  | 3 |  |  | 3 |
|  |  |  | 5.Spur |  |  |  |  | 2 |  |  | 2 |
| 23 | 2017 | upstream | 3.Motherhole |  |  |  | 2 |  |  |  | 2 |
|  |  |  | 4.Vanitys |  |  |  | 2 |  |  |  | 2 |
|  |  |  | 5.Spur |  |  |  |  | 1 |  |  | 1 |
| 24 | 2002-2013 | None | 5.Spur | 5 |  |  |  |  |  |  | 5 |
| 25 | 2017 | None | 2.Condor |  |  | 5 |  |  |  |  | 5 |
| 26 | 2002-2013 | None | 4.Vanitys | 5 |  |  |  |  |  |  | 5 |
| 27 | 2016 | unclear* | 3.Motherhole |  |  |  | 2 |  |  |  | 2 |
|  |  |  | 4.Vanitys |  | 2 |  |  |  |  |  | 2 |
|  |  |  | 5.Spur |  |  |  | 1 |  |  |  | 1 |
| Total for families of 5 or more individuals | | | | 10 | 4 | 11 | 50 | 89 | 79 | 35 | 278 |

**Table E3** Distribution of 49 full-sib families with 2-4 full-sibs across samples and years (Data in ***Cotter_Cataract.genetics.fitness.csv***). Note the addition of column “2013” and deletion of column “Inferred direction of movement” compared to Table E2). The two most upstream sites, Burkes and Pipeline, are highlighted in yellow.

| **FamilyID** | **Cohort** | **Sampling site** | **Year sampled** | | | | | | | | **N inds per site per family** |
| --- | --- | --- | --- | --- | --- | --- | --- | --- | --- | --- | --- |
|  |  |  | 2011 | 2013 | 2016 | 2017 | 2018 | 2019 | 2020 | 2021 |  |
| 28 | 2019 | 4.Vanitys |  |  |  |  |  |  |  | 1 | 1 |
|  |  | 5.Spur |  |  |  |  |  | 1 | 1 | 1 | 3 |
| 29 | 2019 | 2.Condor |  |  |  |  |  | 1 |  |  | 1 |
|  |  | 3.Motherhole |  |  |  |  |  |  | 3 |  | 3 |
| 30 | 2018 | 7.Burkes |  |  |  |  |  | 4 |  |  | 4 |
| 31 | 2018 | 6.Pipeline |  |  |  |  |  |  |  | 1 | 1 |
|  |  | 7.Burkes |  |  |  |  |  | 3 |  |  | 3 |
| 32 | 2018 | 5.Spur |  |  |  |  |  | 2 | 2 |  | 4 |
| 33 | 2017 | 6.Pipeline |  |  |  |  |  | 4 |  |  | 4 |
| 34 | 2016 | 4.Vanitys |  |  | 1 |  |  |  |  |  | 1 |
|  |  | 5.Spur |  |  |  |  | 3 |  |  |  | 3 |
| 35 | 2002-2013 | 4.Vanitys | 3 |  |  |  |  |  |  |  | 3 |
|  |  | 5.Spur | 1 |  |  |  |  |  |  |  | 1 |
| 36 | 2020 | 4.Vanitys |  |  |  |  |  |  |  | 1 | 1 |
|  |  | 5.Spur |  |  |  |  |  |  |  | 2 | 2 |
| 37 | 2020 | 4.Vanitys |  |  |  |  |  |  | 3 |  | 3 |
| 38 | 2016, | 1.Reservoir |  |  |  |  |  | 1 |  |  | 1 |
|  | 2018, | 2.Condor |  |  | 1 |  |  |  |  |  | 1 |
|  | 2019 | 3.Motherhole |  |  |  |  |  | 1 |  |  | 1 |
| 39 | 2019 | 6.Pipeline |  |  |  |  |  | 1 |  | 2 | 3 |
| 40 | 2002-2013 | 4.Vanitys | 2 |  |  |  |  |  |  |  | 2 |
|  |  | 5.Spur | 1 |  |  |  |  |  |  |  | 1 |
| 41 | 2017 | 3.Motherhole |  |  |  |  | 1 |  |  |  | 1 |
|  |  | 4.Vanitys |  |  |  |  | 1 |  |  |  | 1 |
|  |  | 5.Spur |  |  |  |  |  | 1 |  |  | 1 |
| 42 | 2017 | 4.Vanitys |  |  |  |  | 3 |  |  |  | 3 |
| 43 | 2002-2013 | 5.Spur | 2 |  |  |  |  |  |  |  | 2 |
|  |  | 6.Pipeline | 1 |  |  |  |  |  |  |  | 1 |
| 44 | 2016 | 4.Vanitys |  |  | 2 |  |  |  |  |  | 2 |
|  |  | 5.Spur |  |  |  |  | 1 |  |  |  | 1 |
| 45 | 2002-2013 | 1.Reservoir |  | 3 |  |  |  |  |  |  | 3 |
| 46 | 2019 | 4.Vanitys |  |  |  |  |  |  |  | 1 | 1 |
|  |  | 5.Spur |  |  |  |  |  |  | 1 |  | 1 |
| 47 | 2020 | 4.Vanitys |  |  |  |  |  |  |  | 2 | 2 |
| 48 | 2019 | 6.Pipeline |  |  |  |  |  |  |  | 2 | 2 |
| 49 | 2020 | 4.Vanitys |  |  |  |  |  |  | 1 |  | 1 |
|  |  | 5.Spur |  |  |  |  |  |  | 1 |  | 1 |
| 50 | 2020 | 4.Vanitys |  |  |  |  |  |  | 1 | 1 | 2 |
| 51 | 2019 | 5.Spur |  |  |  |  |  | 2 |  |  | 2 |
| 52 | 2019 | 1.Reservoir |  |  |  |  |  | 1 |  |  | 1 |
|  |  | 3.Motherhole |  |  |  |  |  |  | 1 |  | 1 |
| 53 | 2002-2013 | 4.Vanitys | 1 |  |  |  |  |  |  |  | 1 |
|  |  | 5.Spur | 1 |  |  |  |  |  |  |  | 1 |
| 54 | 2017 | 6.Pipeline |  |  |  |  |  | 1 | 1 |  | 2 |
| 55 | 2017 | 6.Pipeline |  |  |  |  |  | 2 |  |  | 2 |
| 56 | 2017 | 4.Vanitys |  |  |  |  | 2 |  |  |  | 2 |
| 57 | 2002-2013 | 4.Vanitys |  | 2 |  |  |  |  |  |  | 2 |
| 58 | 2016 | 7.Burkes |  |  |  |  | 2 |  |  |  | 2 |
| 59 | 2017 | 2.Condor |  |  |  | 2 |  |  |  |  | 2 |
| 60 | 2016 | 6.Pipeline |  |  |  |  | 2 |  |  |  | 2 |
| 61 | 2017 | 4.Vanitys |  |  |  |  | 1 |  |  |  | 1 |
|  |  | 5.Spur |  |  |  |  | 1 |  |  |  | 1 |
| 62 | 2016 | 2.Condor |  |  | 1 |  |  |  |  |  | 1 |
|  |  | 5.Spur |  |  |  |  | 1 |  |  |  | 1 |
| 63 | 2017 | 6.Pipeline |  |  |  |  |  | 2 |  |  | 2 |
| 64 | 2018 | 3.Motherhole |  |  |  |  |  | 1 |  |  | 1 |
|  |  | 4.Vanitys |  |  |  |  |  | 1 |  |  | 1 |
| 65 | 2018 | 3.Motherhole |  |  |  |  |  | 2 |  |  | 2 |
| 66 | 2017 | 4.Vanitys |  |  |  |  | 1 |  |  | 1 | 2 |
| 67 | 2002-2013 & 2016 | 2.Condor |  |  | 1 |  |  |  |  |  | 1 |
|  |  | 4.Vanitys |  | 1 |  |  |  |  |  |  | 1 |
| 68 | 2016 | 6.Pipeline |  |  |  |  | 2 |  |  |  | 2 |
| 69 | 2018 | 2.Condor |  |  |  |  | 1 |  |  |  | 1 |
|  |  | 3.Motherhole |  |  |  |  |  |  | 1 |  | 1 |
| 70 | 2018 | 2.Condor |  |  |  |  | 1 |  |  |  | 1 |
|  |  | 3.Motherhole |  |  |  |  |  | 1 |  |  | 1 |
| 71 | 2018 | 6.Pipeline |  |  |  |  |  | 1 | 1 |  | 2 |
| 72 | 2018 | 3.Motherhole |  |  |  |  |  | 2 |  |  | 2 |
| 73 | 2002-2013 | 5.Spur | 1 | 1 |  |  |  |  |  |  | 2 |
| 74 | 2017 | 2.Condor |  |  |  | 1 |  |  |  |  | 1 |
|  |  | 3.Motherhole |  |  |  |  | 1 |  |  |  | 1 |
| 75 | 2017 | 3.Motherhole |  |  |  |  | 2 |  |  |  | 2 |
| 76 | 2016 | 6.Pipeline |  |  |  |  | 2 |  |  |  | 2 |
| Total for all families of 2 or more inds (inc. Table E2) | | | 23 | 7 | 10 | 14 | 78 | 124 | 96 | 50 | 402 |

**Appendix S6.** Analyses of heterozygosity and inbreeding depression (R script ***3.Analyses_of_genetics_and_lenth.residuals_incl_ID.R***, data ***Cotter_Cataract.genetics.finess.csv***).

**Table F1.** Mean (SD) individual heterozygosity (PHt) estimated from complete Cotter+Cataract dataset, per multi-age group/cohort and Cataract translocation year (boxplots on Figure F1).

| **Waterbody** | **Cohort** | **Mean PHt** | **SD PHt** |
| --- | --- | --- | --- |
| Cataract Dam | 2017 | 0.1333 | 0.0159 |
| Cataract Dam | 2018 | 0.1575 | 0.0075 |
| Cataract Dam | 2019 | 0.1397 | 0.0069 |
| Cotter River | 2002-2013 | 0.0906 | 0.0099 |
| Cotter River | 2016 | 0.0912 | 0.0083 |
| Cotter River | 2017 | 0.0901 | 0.0097 |
| Cotter River | 2018 | 0.0840 | 0.0113 |
| Cotter River | 2019 | 0.0887 | 0.0169 |
| Cotter River | 2020 | 0.0879 | 0.0096 |

**Table F2.** Results of linear models (LM) built for Cotter-only individuals born 2016-onwards, testing relationships between individual heterozygosity (PHt, proportion of heterozygous sites) and birth-year, cohort distance to Cotter Reservoir and age at capture.

| **Model** | **DF** | **Response** | **Predictors** | **Estimate** | **P-value** | **Adjusted R-squared** |
| --- | --- | --- | --- | --- | --- | --- |
| LM1a | 521 | PHt | Birth-year (continuous) | -0.0015 | **0.0006** | 0.0204 |
| LM1b | 518 | PHt | Cohort (category) | See  Fig. F2A* | **3.8e-08** | 0.0675 |
| LM2 | 521 | PHt | Distance to Cotter | -0.0009 | **1.2e-07** | 0.0506 |
| LM3 | 451 | PHt | Age at capture | -0.0014 | 0.125 |  |
| LM4 | 451 | Distance to Cotter | Age at capture | 0.0542 | **2.0e-10** | 0.0839 |

***Table F2a.** Results of post-hoc Tukey’s test for LM1b

| Pair compared | diff | lwr | upr | p adj |
| --- | --- | --- | --- | --- |
| 2017-2016 | -0.0014 | -0.0061 | 0.0034 | 0.9312 |
| **2018-2016** | -0.0087 | -0.0130 | -0.0044 | **0.0000** |
| **2019-2016** | -0.0051 | -0.0097 | -0.0004 | **0.0237** |
| 2020-2016 | -0.0041 | -0.0101 | 0.0019 | 0.3356 |
| **2018-2017** | -0.0073 | -0.0113 | -0.0033 | **0.0000** |
| 2019-2017 | -0.0037 | -0.0080 | 0.0006 | 0.1373 |
| 2020-2017 | -0.0027 | -0.0085 | 0.0031 | 0.6986 |
| 2019-2018 | 0.0036 | -0.0002 | 0.0075 | 0.0761 |
| 2020-2018 | 0.0046 | -0.0008 | 0.0100 | 0.1431 |
| 2020-2019 | 0.0010 | -0.0047 | 0.0067 | 0.9906 |

**Table F3.** Inbreeding depression models (LM6 - linear model, LMM6 - linear mixed model), for all Cotter-only individuals born 2016-onwards (LM6 is plotted on Fig. F3), and for each of the five cohorts (LM6-2018 is plotted on Fig. 4 of the main ms).

| **Model** | **Random effect** | **DF** | **N obs** | **N groups** | **Response** | **Predictor** | **Esti-mate** | **P-value** | **Adjusted R^2^** |
| --- | --- | --- | --- | --- | --- | --- | --- | --- | --- |
| LM5 | NA | 438 |  |  | Growth residuals | Cohort | See  Fig. B2* | <2.2e-16 | 0.2458 |
| LM6 | NA | 439 |  |  | Growth residuals | PHt | -56.61 | 0.098 | NA |
| LMM6 | Cohort | 435 | 441 | 5 | Growth residuals | PHt | 63.70 | **0.0432** |  |
| LM6-2016 | NA | 81 |  |  | Growth residuals | PHt | 119.10 | 0.1815 | NA |
| LM6-2017 | NA | 97 |  |  | Growth residuals | PHt | 5.10 | 0.948 | NA |
| LM6-2018 | NA | 144 |  |  | Growth residuals | PHt | 158.75 | **0.0013** | 0.0630 |
| LM6-2019 | NA | 67 |  |  | Growth residuals | PHt | -28.96 | 0.706 | NA |
| LM6-2020 | NA | 42 |  |  | Growth residuals | PHt | -115.96 | 0.0843 | NA |

***Table F3a**. Results of post-hoc Tukey’s test for LM5

| Pair compared | diff | lwr | upr | p adj |
| --- | --- | --- | --- | --- |
| 2017-2016 | -1.5687 | -4.6663 | 1.5290 | 0.6363 |
| **2018-2016** | 8.9109 | 6.0497 | 11.7721 | **0.0000** |
| **2019-2016** | 5.5328 | 2.1420 | 8.9237 | **0.0001** |
| 2020-2016 | 0.2658 | -3.6156 | 4.1471 | 0.9997 |
| **2018-2017** | 10.4795 | 7.7697 | 13.1894 | **0.0000** |
| **2019-2017** | 7.1015 | 3.8374 | 10.3656 | **0.0000** |
| 2020-2017 | 1.8344 | -1.9367 | 5.6056 | 0.6711 |
| **2019-2018** | -3.3781 | -6.4187 | -0.3374 | **0.0208** |
| **2020-2018** | -8.6451 | -12.2246 | -5.0656 | **0.0000** |
| **2020-2019** | -5.2670 | -9.2825 | -1.2516 | **0.0033** |

**Figure F1.** Boxplots of individual heterozygosity (PHt, proportion of heterozygous sites) of the three sets of the Cataract Dam individuals translocated to the Cotter River in 2017, 2018 and 2019, the multi-age group (2002-2013) and five Cotter cohorts estimated from the Cotter+Cataract dataset. The two highly heterozygous individuals for Cotter 2019 cohort are admixed individuals. Boxes represent the distribution of the data between the 25th and 75th percentiles, and line in the box is a median.


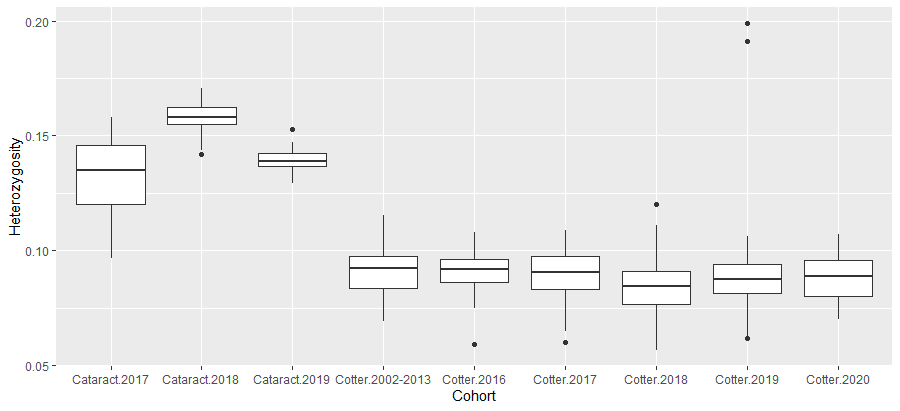


**Figure F2**. Plots of linear models for Cotter-only (no admixed) individuals: A – lowest mean individual heterozygosity (PHt) in 2018 cohort (Tukey’s tests in Table F2a); B - decline of PHt with distance to Cotter Reservoir; C – no relationship between PHt and age at sampling, D - increase of distance to Cotter Reservoir with age at sampling. The actual distance from Cotter Reservoir to Condor, assumed here to be 0.3 km, depends on the reservoir depth.


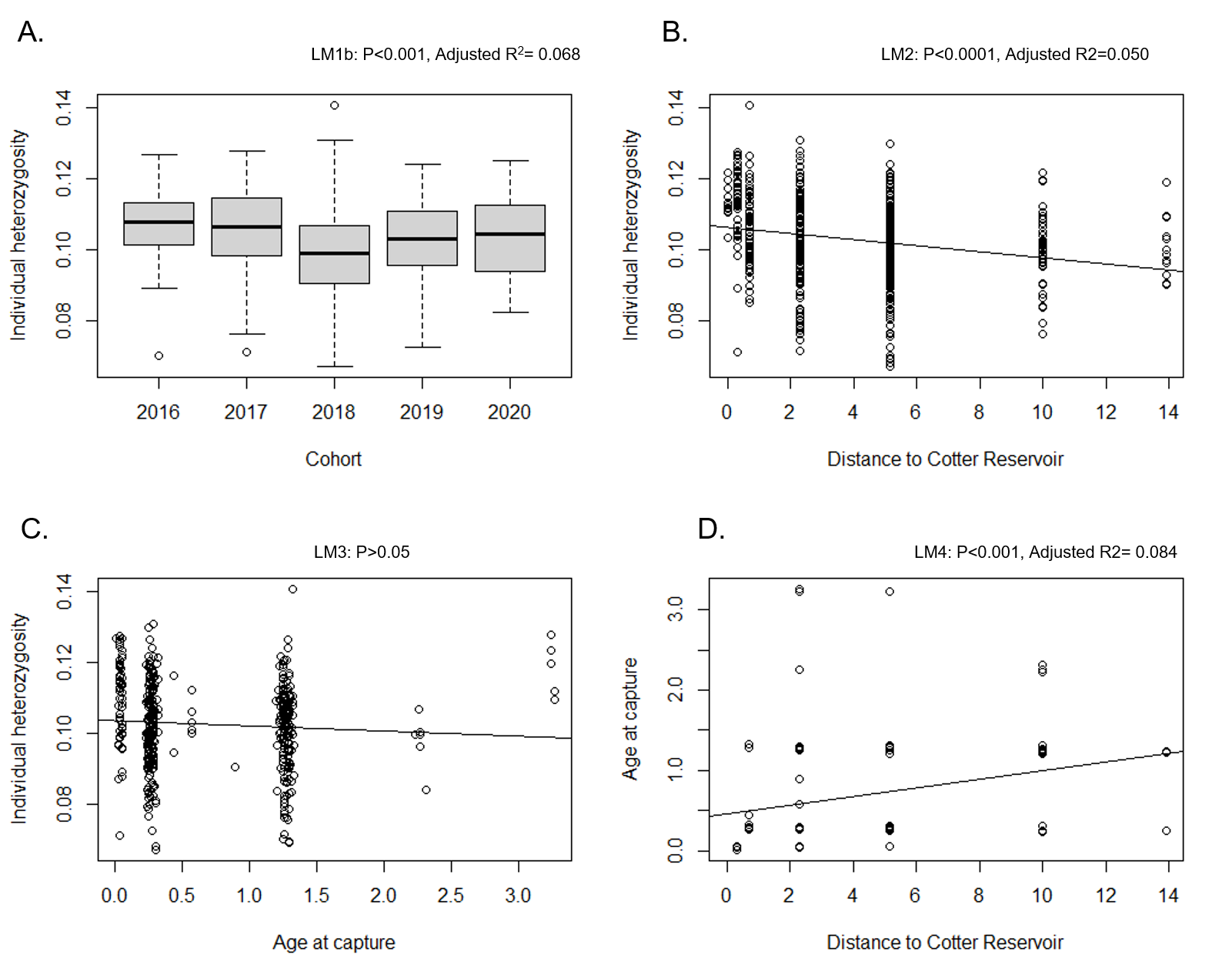


**Figure F3.** Scatterplot of growth residuals vs heterozygosity for all Cotter cohorts.


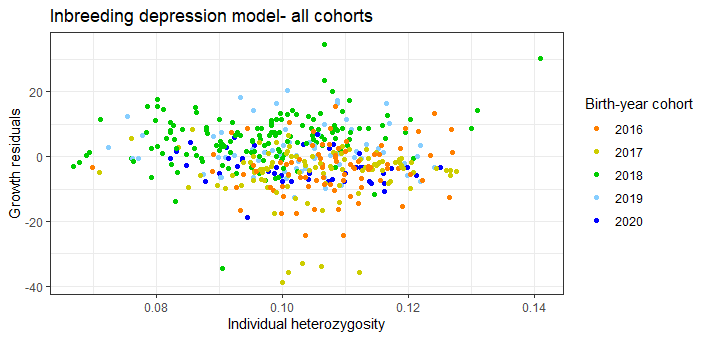


**Appendix S7.** Analyses of effective population sizes (correlation between flow and Ne estimates done in R script ***4.Analyses_of_flow_Ne_disp.dist.R***, data in ***Flow_vs_NE.csv***).

**Table G1.** Results of *Colony2* Ne (Nb) analyses for the multi-age group (2002-2013) and each cohort using more stringently filtered dataset (i.e. as for parentage analyses). Numbers in parenthesis are lower and upper 95% confidence intervals.

|  | **Cohorts of Cotter fish** | | | | | |
| --- | --- | --- | --- | --- | --- | --- |
| **Parameters** | **2002-2013** | **2016** | **2017** | **2018** | **2019** | **2020** |
| All sites; no admixed | 45  (30-69) | 34  (23-57) | 23  (14-42) | 20  (12-39) | 16  (9-34) | 13  (7-30) |
| All sites; with admixed |  |  |  |  | 17  (10-34) |  |
| Spur+Vanitys; no admixed | 22  (13-39) | 20  (12-38) | 12  (7-28) | 9  (5-28) | 9  (5-24) | 13  (7-30) |
| Spur+Vanitys; with admixed |  |  |  |  | 9  (4-24) |  |

*N*e estimated by *Colony2* from all Cotter-only data during parentage analysis, averaged across five replicates, was 61 (44-87).

**Literature cited**

Broadhurst, B.T., Clear, R.C., Lintermans, M., 2016. Potential barriers to upstream migration of Macquarie perch to spawning areas in the Cotter River, ACT. Institute for Applied Ecology, University of Canberra, Canberra.

Broadhurst, B.T., Lintermans, M., Clear, R.C., van der Meulen, D., 2020. Spawning movements of Macquarie perch in the Cotter River 2019. Report to Icon Water. Institute for Applied Ecology, University of Canberra, Canberra.

Ebner, B., Lintermans, M., 2007. Fish passage, movement requirements and habitat use for Macquarie perch. Final report to the Department of Agriculture, Fisheries and Forestry Australia. Parks, Conservation and Lands, Canberra, 139.

Ebner, B., Thiem, J., Broadhurst, B., Clear, R., Frawley, K., 2008. Delivering environmental flows to large biota. Final Report to the Department of the Environment, Water, Heritage and the Arts. Parks, Conservation and Lands, Canberra.

Tonkin, Z., Kearns, J., Lyon, J., Balcombe, S.R., King, A.J., Bond, N.R., 2017. Regional-scale extremes in river discharge and localised spawning stock abundance influence recruitment dynamics of a threatened freshwater fish. Ecohydrology 10.
