# Supplementary material for: Planning and implementing genetic rescue of an endangered freshwater fish population in a regulated river, where low flow reduces breeding opportunities and may trigger inbreeding depression
Source: Evol Appl. 2024 Apr 11;17(4):e13679. doi: 10.1111/eva.13679 (PMC11009430; doi:10.1111/eva.13679)
Supplement: Supplementary file 2 — Data S1 [file EVA-17-e13679-s001.docx]

**Supplementary Material S1 for the manuscript**

Pavlova, A., Schneller, N., Lintermans, M., Beitzel, M., Robledo-Ruiz, D., Sunnucks, P. Planning and implementing genetic rescue of an endangered freshwater fish population in a regulated river, where low flow reduces breeding opportunities and may trigger inbreeding depression. *Evolutionary Applications* DOI: 10.1111/EVA.13679.

In response to the Associate Editor’s comment

“*the test for inbreeding depression could be improved if the authors analyzed a single model with all cohorts and single factors such as site, flow, individual inbreeding plus interactions included in the model. Presumably the interaction between flow and inbreeding would emerge as a significant predictor of body size.*”

we attempted to fit an interaction between PHt and cohort (PHt*cohort) in the inbreeding depression model (Table S1).

A forward stepwise model selection process was used to explore whether more complex models explained significantly more variance in the data. We started by fitting a linear model (LM5) testing whether cohorts differed in growth residuals (growth residuals ~ cohort). Next, to test for inbreeding depression, we first tested PHt as a single predictor of growth residuals (LM6: growth residuals ~ PHt). Second, we included both PHt and cohort as fixed predictors (LM7: growth residuals ~ PHt + cohort) in order to control for differences in growth residuals due to the year in which each cohort was born. Given that model LM7 assumes that the slopes (i.e., strength of inbreeding depression) are not different across cohorts, this model was unable to test out prediction of a stronger inbreeding depression (i.e. more positive slope) for more stressful years. Therefore, we built another model in which we tested whether the strength of inbreeding depression (slope of the model) differed among cohorts by testing the interaction of PHt and cohort (LM8: growth residuals ~ PHt * cohort). ANOVA was used to compare models and determine whether adding new predictors significantly improved the fit. In addition, we attempted to fit the most complex model suggested by the Associate Editor LM9: growth_residuals ~ PHt * cohort + site, except we were unable to include flow as a predictor because all members of the same cohort had the same value of flow and thus, flow and cohort were redundant terms.

Cohort alone significantly explained 24.7% of variance in growth residuals (LM5 P<0.001, R^2^ = 0.247), while individual heterozygosity alone did not predict growth residuals (LM6 P>0.05). Including both heterozygosity and cohort as a fixed effect (LM7 P=0.043, R^2^=0.253) significantly improved the fit of the model, compared to both single-predictor models (ANOVA test LM7 vs LM5 P=0.034, LM7 vs LM6 P<0.001). Growth residuals significantly increased with heterozygosity when cohort was controlled for (LM7), indicating that more-inbred (less heterozygous) individuals grow slower than less-inbred (more heterozygous) ones. However, including the interaction of individual heterozygosity and cohort (LM8 P<0.001, R^2^=0.26) did not significantly improve the fit of the model (ANOVA test LM8 vs LM 7 P = 0.054) and none of the model terms were significant. When we fitted PHt * cohort + site as predictors (LM9 P<0.001, R^2^=0.30), site7.Burkes (which is the most upstream site) emerged as the site with significantly lower growth residuals, and the only significant predictor of growth residuals.

As a post-hoc analysis, we split the data into cohorts to test for inbreeding depression in each of them individually (LM6-2016 to LM6-2020: growth residuals ~ PHt).

**Table S1.** Results of the models built during forward stepwise model selection.

|  | Estimate | Std. error | DF | t-value | P-value | R^2^ |
| --- | --- | --- | --- | --- | --- | --- |
| **LM5: growth_residuals ~ cohort** | | | | | | 0.247 |
| Intercept | -3.69 | 0.83 | 439 | -4.42 | **1.24e-05** |  |
| Cohort2017 | -1.57 | 1.13 | 439 | -1.39 | 0.166 |  |
| Cohort2018 | 8.91 | 1.04 | 439 | 8.53 | **2.42e-16** |  |
| Cohort2019 | 5.53 | 1.24 | 439 | 4.47 | **1.00e-05** |  |
| Cohort2020 | 0.27 | 1.42 | 439 | 0.19 | 0.851 |  |
| **LM6: growth_residuals ~ PHt** | | | | | | 0.004 |
| Intercept | 5.6 | 3.52 | 439 | 1.59 | 0.113 |  |
| PHt | -56.61 | 34.17 | 439 | -1.66 | 0.908 |  |
| **LM7: growth_residuals ~ PHt + cohort** | | | | | | 0.253 |
| Intercept | -10.86 | 3.48 |  | -13.12 | **0.002** |  |
| PHt | 66.78 | 31.47 |  | 2.12 | **0.034** |  |
| Cohort2017 | -1.45 | 1.13 |  | -1.28 | 0.20 |  |
| Cohort2018 | 9.61 | 1.09 |  | 8.80 | **< 2e-16** |  |
| Cohort2019 | 5.86 | 1.24 |  | 4.72 | **3.19e-06** |  |
| Cohort2020 | 0.54 | 1.42 |  | 0.38 | 0.70 |  |
| **LM8:** **growth_residuals ~ PHt * cohort** | | | | | | 0.2625 |
| Intercept | -16.48 | 9.22 | 431 | -1.79 | 0.075 |  |
| PHt | 119.09 | 85.47 | 431 | 1.39 | 0.164 |  |
| Cohort2017 | 10.69 | 11.78 | 431 | 0.91 | 0.365 |  |
| Cohort2018 | 6.32 | 10.38 | 431 | 0.61 | 0.543 |  |
| Cohort2019 | 21.29 | 12.30 | 431 | 1.73 | 0.084 |  |
| Cohort2020 | 25.04 | 14.06 | 431 | 1.78 | 0.076 |  |
| PHt*Cohort2017 | -113.99 | 109.92 | 431 | -1.04 | 0.300 |  |
| PHt*Cohort2018 | 39.66 | 98.42 | 431 | 0.40 | 0.687 |  |
| PHt*Cohort2019 | -148.05 | 116.37 | 431 | -1.27 | 0.204 |  |
| PHt*Cohort2020 | -235.05 | 133.15 | 431 | -1.77 | 0.078 |  |
| **LM9: growth_residuals ~ PHt * cohort + site** | | | | | | 0.2998 |
| Intercept | -10.62 | 9.44 |  | -1.13 | 0.261 |  |
| PHt | 91.92 | 84.41 |  | 1.09 | 0.277 |  |
| Cohort2017 | 9.28 | 11.54 |  | 0.80 | 0.422 |  |
| Cohort2018 | 5.53 | 10.25 |  | 0.54 | 0.590 |  |
| Cohort2019 | 18.62 | 12.06 |  | 1.54 | 0.123 |  |
| Cohort2020 | 23.80 | 13.80 |  | 1.73 | 0.085 |  |
| 3.Motherhole | -0.76 | 1.83 |  | -0.42 | 0.677 |  |
| 4.Vanitys | -1.21 | 1.72 |  | -0.71 | 0.481 |  |
| 5.Spur | -3.38 | 1.73 |  | -1.95 | 0.052 |  |
| 6.Pipeline | -3.49 | 1.96 |  | -1.78 | 0.075 |  |
| 7.Burkes | -10.66 | 2.51 |  | -4.24 | **2.7e-05** |  |
| PHt*Cohort2017 | -116.25 | 107.70 |  | -1.08 | 0.281 |  |
| PHt*Cohort2018 | 45.41 | 97.47 |  | 0.47 | 0.642 |  |
| PHt*Cohort2019 | -125.30 | 114.23 |  | -1.10 | 0.273 |  |
| PHt*Cohort2020 | -233.42 | 131.05 |  | -1.78 | 0.076 |  |

The R code for the analyses described above is *ID_models_with_interaction.R*, and the data is in *Cotter_Cataract.genetics.finess.csv* are available on Bridges data repository at <https://doi.org/10.26180/19376570>.
